# Bacterial alterations in salivary microbiota and their association in oral cancer

**DOI:** 10.1038/s41598-017-16418-x

**Published:** 2017-11-28

**Authors:** Wei-Hsiang Lee, Hui-Mei Chen, Shun-Fa Yang, Chao Liang, Chih-Yu Peng, Feng-Mao Lin, Lo-Lin Tsai, Buor-Chang Wu, Chung-Han Hsin, Chun-Yi Chuang, Ting Yang, Tzu-Ling Yang, Shinn-Ying Ho, Wen-Liang Chen, Kwo-Chang Ueng, Hsien-Da Huang, Chien-Ning Huang, Yuh-Jyh Jong

**Affiliations:** 1 Department of Medical Research, Chung Shan Medical University Hospital, Taichung, 402 Taiwan; 2 Department of Biological Science and Technology, National Chiao Tung University, Hsinchu, 300 Taiwan; 3 Institute of Bioinformatics and Systems Biology, National Chiao Tung University, Hsinchu, 300 Taiwan; 4 Institute of Medicine, Chung Shan Medical University, Taichung, 402 Taiwan; 5 School of Dentistry, Chung Shan Medical University, Taichung, 402 Taiwan; 6 Department of Dentistry, Chung Shan Medical University Hospital, Taichung, 402 Taiwan; 7 School of Medicine, Chung Shan Medical University, Taichung, 402 Taiwan; 8 Department of Otolaryngology, Chung Shan Medical University Hospital, Taichung, 402 Taiwan; 9 Department of Internal Medicine, Division of Endocrinology and Metabolism, Chung Shan Medical University Hospital, Taichung, 402 Taiwan; 10 Graduate Institute of Clinical Medicine, College of Medicine, Kaohsiung Medical University, Kaohsiung, 807 Taiwan; 11 Departments of Paediatrics and Laboratory Medicine, Kaohsiung Medical University Hospital, Kaohsiung Medical University, Kaohsiung, 807 Taiwan

## Abstract

Oral squamous cell carcinoma (OSCC) is the most common malignant neoplasm of the oral cavity and the fourth leading malignancy and cause of cancer-related death in the male population of Taiwan. Most cases are detected at advanced stages, resulting in poor prognosis. Therefore, improved detection of early oral health disorders is indispensable. The involvement of oral bacteria in inflammation and their association with OSCC progression provide a feasible target for diagnosis. Due to the nature of oral neoplasms, the diagnosis of epithelial precursor lesions is relatively easy compared with that of other types of cancer. However, the transition from an epithelial precursor lesion to cancer is slow and requires further and continuous follow-up. In this study, we investigated microbiota differences between normal individuals, epithelial precursor lesion patients, and cancer patients with different lifestyle habits, such as betel chewing and smoking, using next-generation sequencing. Overall, the oral microbiome compositions of five genera, *Bacillus*, *Enterococcus*, *Parvimonas*, *Peptostreptococcus*, and *Slackia*, revealed significant differences between epithelial precursor lesion and cancer patients and correlated with their classification into two clusters. These composition changes might have the potential to constitute a biomarker to help in monitoring the oral carcinogenesis transition from epithelial precursor lesion to cancer.

## Introduction

Oral cancer ranks sixth among the most common cancers worldwide, with specifically high prevalence rates in Europe, Melanesia, and Southcentral Asia^1^. In 2011, the 5-year survival rate for oral cancer patients was only 60% in the United States^2^, and it became the sixth most common cancer in Taiwan and the fourth most common cancer among Taiwanese males^3^. Despite recent advances in the multidisciplinary treatment of oral cancer, the prognosis of patients with locally advanced disease and the relapse rate of disease-free patients remain unsatisfactory^4^. Therefore, an improved understanding of the cellular and molecular events that initiate oral tumours and promote metastasis is being pursued^5^.

Oral habits, such as betel quid chewing, alcohol consumption, and cigarette smoking, have been documented as risk factors for oral cancer^6,7^. Reports have confirmed transitions from epithelial precursor lesion (leukoplakia, oral submucosal fibrosis, and others) to cancer over time^8–10^. Accordingly, habitual cigarette smokers, alcohol consumers, and betel quid chewers should receive regular oral screening so that potential oral cancer and epithelial precursor lesions can be detected as early as possible. *Helicobacter pylori* is the first conventionally recognised bacterial carcinogen^11^; another correlation between pathogenic bacteria and oral disease is *Porphyromonas gingivalis* infection and periodontitis^12^. Moreover, oral microbiota alterations have been detected in alcohol^13^, betel^14^, and cigarette^15,16^ consumers. Hence, the link between oral cancer-inducing factors and bacterial ecology should not be neglected.

Most oral cancers are oral squamous cell carcinomas (OSCCs)^17^. The association of bacteria with OSCC progression can be explained by inflammation-induced DNA damage in epithelial cells caused by microorganism-secreted endotoxins^18,19^. Scientists searched for a correlation between microbes and oral cancer even before the availability of high-throughput technologies. Some specific species have been identified to correlate strongly with OSCC, such as *Porphyromonas gingivalis*, *Fusobacterium nucleatum*, and *Prevotella intermedia* ^20–24^. Researchers have also found that specific bacterial taxa, such as *Veillonella*, *Fusobacterium*, *Prevotella*, *Porphyromonas*, *Actinomyces*, *Clostridium*, *Haemophilus*, *Enterobacteriaceae*, and *Streptococcus* spp., are linked to oral cancer and epithelial precursor lesions^25^. Another work identified increased abundance levels of *Capnocytophaga gingivalis*, *Prevotella melaninogenica*, and *Streptococcus mitis* in the saliva of individuals with OSCC^23,26^. Hooper *et al*. (2007) reported 52 different bacterial phylotypes from tumour tissues using fluorescent *in situ* hybridisation and 16S rRNA sequencing, including *Proteobacteria*, *Fusobacterium*, *Streptococcus*, *Prevotella*, and *Veillonella* ^27^. Thus, the salivary microbiota could be a diagnostic indicator of patients with OSCC compared with OSCC-free controls^24,28^.

Although some species are correlated with oral cancer, the complexity of the cancer-driving conditions associated with the microbiome remains unexplained by a single pathogen. High-throughput assays such as next-generation sequencing (NGS) offer comprehensive culture-free techniques for surveying human microbiome composition and biomolecular activity at the transcriptional level^29–32^. Studies have used NGS to obtain complete (cultured and uncultured) bacterial profiles in OSCC subjects and have identified enrichments of the species *Peptostreptococcus stomatis* and *Streptococcus gordonii* at tumour sites^33^ and significantly lower abundance levels of Firmicutes (especially *Streptococcus*) and Actinobacteria (especially *Rothia*) in cancer samples^34^.

Saliva is in direct contact with oral cancer and epithelial precursor lesions; hence, abnormal DNA, RNA, and protein molecules released by malignant cells can be easily obtained from saliva. The use of saliva as a diagnostic target may avoid unnecessary biopsies and hospital and outpatient clinical visits; it offers an inexpensive, non-invasive, and easily accessible early detection and prognosis tool, and it allows for monitoring post-therapy in oral cancer patients^32,35–37^. We propose to combine NGS and bioinformatics with a less invasive method of collecting reliable specimens and to use changes in oral microbiome composition as an alert regarding oral health disorders that might lead to oral cancer.

## Results

### Characteristics of the study population

A total of 376 individuals were enrolled in the study. Based on diagnostic results, they were classified into three groups: Normal (127), Epithelial precursor lesions (including dysplasia, hyperplasia, and hyperkeratosis^38,39^; (124), and Cancer (125). Individuals from the Normal group showed neither epithelial precursor lesions nor oral cancer, those in the Epithelial precursor lesion group showed epithelial precursor lesions, and those in the Cancer group had OSCC. For the sake of convenience, in this study, the terms “normal individuals” and “healthy individuals” are employed interchangeably. In addition, non-smokers and non-chewers were defined as individuals who had no history of cigarette smoking and betel quid chewing, respectively, prior to having their saliva samples taken. The patient history in terms of TNM classification for cancer patients is presented in Supplementary Tables S1 and S2. The diagnostic results, together with the betel quid chewing and cigarette smoking statuses, are presented in Table 1. The number of both non-smokers and non-chewers was 85, of whom 72.9% (62/85) were normal, 16.5% (14/85) were patients with epithelial precursor lesions, and 10.6% (9/85) had OSCC (Table S3). The number of current chewers who were non-smokers was only five; most subjects were former or current chewers/smokers (Tables S4 and S5). For example, the number of former or current chewers was 230, of whom 15.2% (35/230) were normal, 36.1% (83/230) were patients with epithelial precursor lesions, and 48.7% (112/230) had OSCC. Of the 83 former/current chewers in the Epithelial precursor lesion group, 67.5% (n = 56) were current chewers, whereas of the 112 former/current chewers in the Cancer group, up to 82% were former chewers (n = 91) (Table S3). Hence, the proportions of current or former smokers/chewers in the Epithelial precursor lesion and Cancer groups were high. We also found that both non-smokers and non-chewers in the Cancer and Epithelial precursor lesion groups tended to be older (Fig. S1). For the sake of convenience, the following symbols are used to indicate the patient groups: B−, non-chewer; B*, former chewer; B+, current chewer; C−, non-smoker; C*, former smoker; and C+, current smoker. Based on the number of persons and their betel quid chewing and cigarette smoking statuses, each group was classified into six subgroups: B−C−, B−C*, B−C+, B* C*, B* C+, and B+C+.

**Table 1:** Descriptive characteristics of the study population.

		Normal (n = 127)	Epithelial precursor lesion (n = 124)	Cancer (n = 125)
	Age (years)	52 ± 14	50 ± 11	53 ± 10
	Sex (Male/Female)	117/10	110/14	113/12
Betel Quid Chewing Status	Current chewer (10Y+)	13 (10%)	20 (16%)	16 (13%)
	Current chewer (10Y−)	4 (3%)	7 (6%)	5 (4%)
	Former chewer	18 (14%)	56 (45%)	91 (73%)
	Non-chewer	92 (72%)	41 (33%)	13 (10%)
Cigarette Smoking Status	Current smoker (10Y+)	33 (26%)	72 (58%)	63 (50%)
	Current smoker (10Y−)	4 (3%)	5 (4%)	2 (2%)
	Former smoker	27 (21%)	27 (22%)	39 (31%)
	Non-smoker	63 (50%)	20 (16%)	21 (17%)

### Five predominant phyla in saliva samples

Based on the available samples, 15,392,451 sequencing reads were aligned, identifying an average of 40,937 sequencing reads per sample. We sorted the sequences into 548 operational taxonomic units (OTUs, ≥ 97% ID). Although microbial variability at the phylum level cannot explain individual differences, we could roughly distinguish different microbiotas within the subgroups (Fig. S2). In our samples, the dominant bacterial species belonged to one of five phyla: Firmicutes, Bacteroidetes, Proteobacteria, Actinobacteria, and Fusobacteria. These phyla were present in all individuals, with minor variations among groups. Firmicutes was the predominant phylum in each subgroup, and Bacteroidetes was the second most abundant phylum in most subgroups. Coincidentally, Bacteroidetes displayed the same relative abundance levels within the B−C− subgroup in all three patient groups. In addition, the relative abundance levels of Firmicutes, Bacteroidetes, and Proteobacteria were similar in the B−C* subgroup of Epithelial precursor lesion patients.

### α-Diversity at the genus level

**Figure 1:**
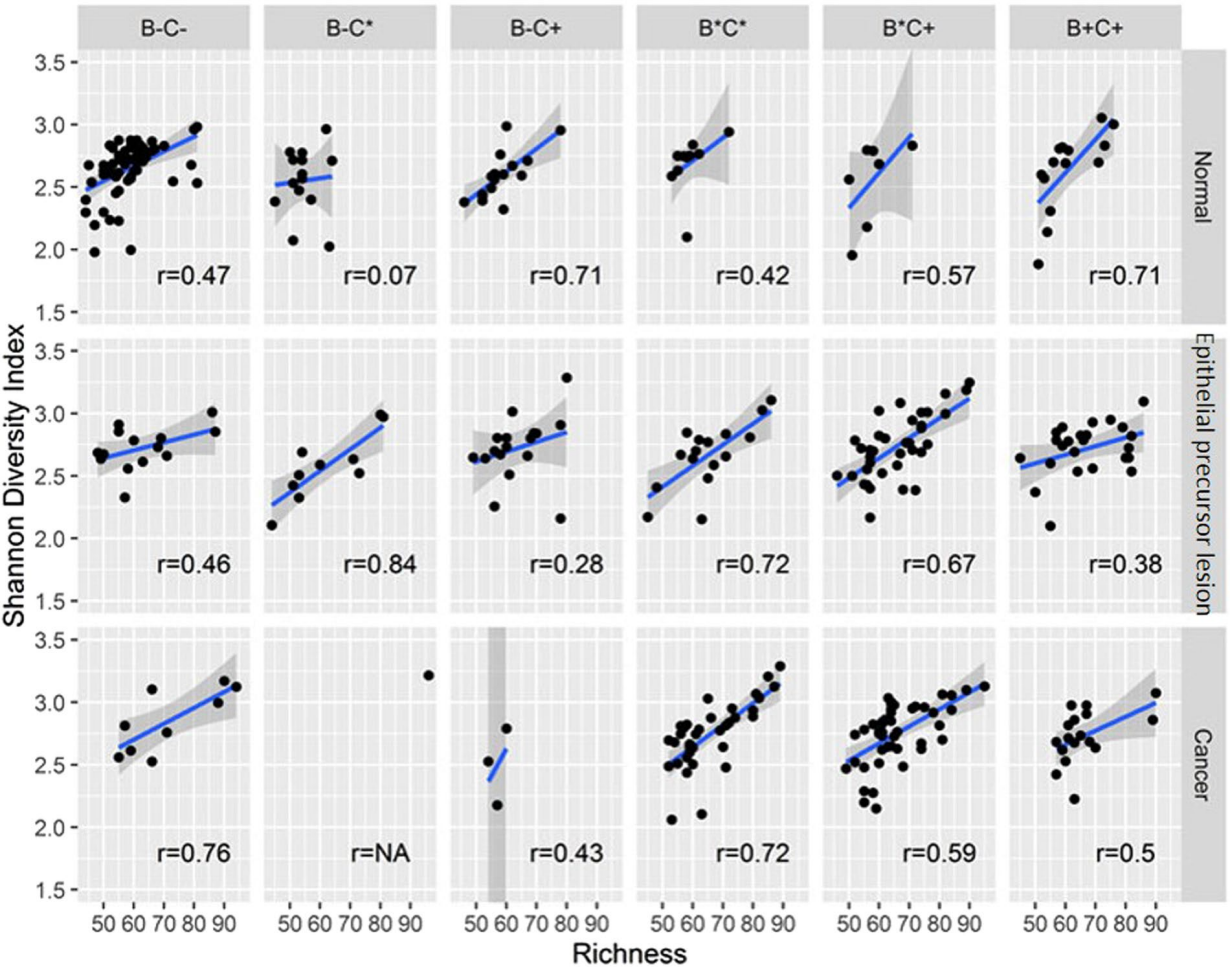
Correlations between species richness and Shannon diversity index within subgroups. In the B+C+ subgroup, the coefficient is as high as 0.7 for the Normal group, whereas it declines to 0.37 and 0.48 for patients with epithelial precursor lesions and OSCC, respectively. The B−C+ subgroup shows a similar pattern. In the B* C* subgroup, the coefficients are 0.42 for the Normal group and 0.7 for the other subgroups. In the B−C* subgroup, the correlation between species richness and the Shannon diversity index for the Normal group is very weak.

To summarise the diversity of the genera constituting the individuals’ microbiotas, we measured the species richness (the total number of species in the community) and the Shannon diversity index. Generally, the Shannon diversity index increases as species richness increases. In our samples, the correlation coefficient between species richness and the Shannon diversity index within subgroups varied widely (Fig. 1). For example, in the B+C+ subgroup, the coefficient was as high as 0.7 for the Normal group, whereas it declined to 0.37 and 0.48 for patients with epithelial precursor lesions and OSCC, respectively. The B−C+ subgroup showed a similar pattern. In the B* C* subgroup, the coefficients were 0.42 for healthy controls and 0.7 for the other subgroups. Interestingly, in the B−C* subgroup, the correlation between species richness and the Shannon diversity index for healthy controls was very weak. In contrast, the correlation coefficient for Epithelial precursor lesion patients was as high as 0.8. Given that there was only one non-chewer and former smoker in the Cancer group, we could not determine the correlation between species richness and the Shannon diversity index. There were no statistically significant differences between any subgroups in the Normal and Epithelial precursor lesion groups except for species richness between the B−C− and B−C* subgroups in the Normal group (Table S6).

Considering that the Shannon diversity index within groups was approximately 2.5, the bacterial communities within individuals’ saliva samples were dominated by approximately 13 genera. We selected the top 15 genera from the average sum of relative abundance in the three groups and preliminarily observed their relationships (Fig. S3). The three groups shared the same top 14 genera, arranged in slightly different orders: *Prevotella*, *Veillonella*, *Streptococcus*, *Neisseria*, *Rothia*, *Fusobacterium*, *Haemophilus*, *Actinomyces*, *Leptotrichia*, *Porphyromonas*, *Bacillus*, *Selenomonas*, *Carnobacterium*, and *Eubacterium*. Among these genera, *Prevotella* was the most predominant in all groups. The fifteenth genus corresponded to *Megasphaera*, *Escherichia*, and *Atopobium* in the Normal, Epithelial precursor lesion, and Cancer groups, respectively.

Based on cigarette smoking and betel quid chewing statuses, the distribution of the top 15 genera differed slightly within the groups (Fig. S3). For instance, in the Normal group, the relative abundance of *Prevotella* was lower in the B* C* subgroup than in the other subgroups, whereas in the B* C+ subgroup, the opposite was true. According to principal component analysis based on the top 10 genera, there was no separate clustering between the groups (Fig. S4). Nevertheless, within non-chewers, the correlation between *Prevotella* and the other genera was almost negative in the Normal group (Fig. S5), whereas *Prevotella* was positively correlated with *Leptotrichia* in the Epithelial precursor lesion group (Fig. S6). Furthermore, strong positive correlations were evident between *Prevotella* and *Veillonella* and between *Prevotella* and *Leptotrichia* within both current smokers and chewers (Fig. S7). Further conditions are presented in Supplementary Figure S8. Microbiota compositions at the genus level within individuals’ saliva samples are presented in Supplementary Figures S9–S11.

### Biodiversity comparisons between groups and relationships between specific genera

**Figure 2:**
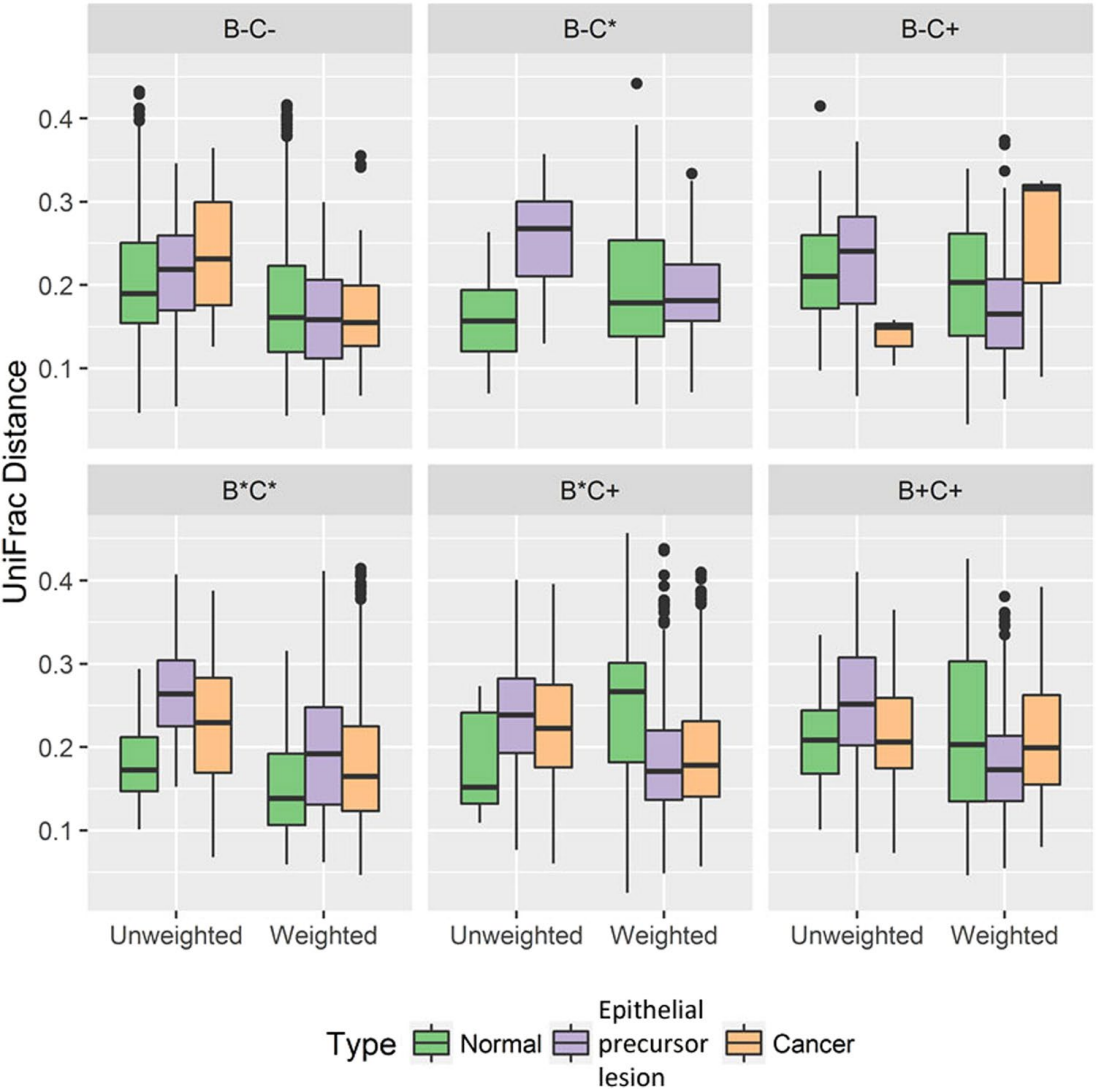
Boxplots of UniFrac (unweighted and weighted) distances between salivary microbial communities using the entire phylogenetic tree. Most conditions revealed statistically significant differences between any two subgroups, particularly for the B* C+ subgroup, in which p values for unweighted or weighted UniFrac distances were all less than 0.03.

**Figure 3:**
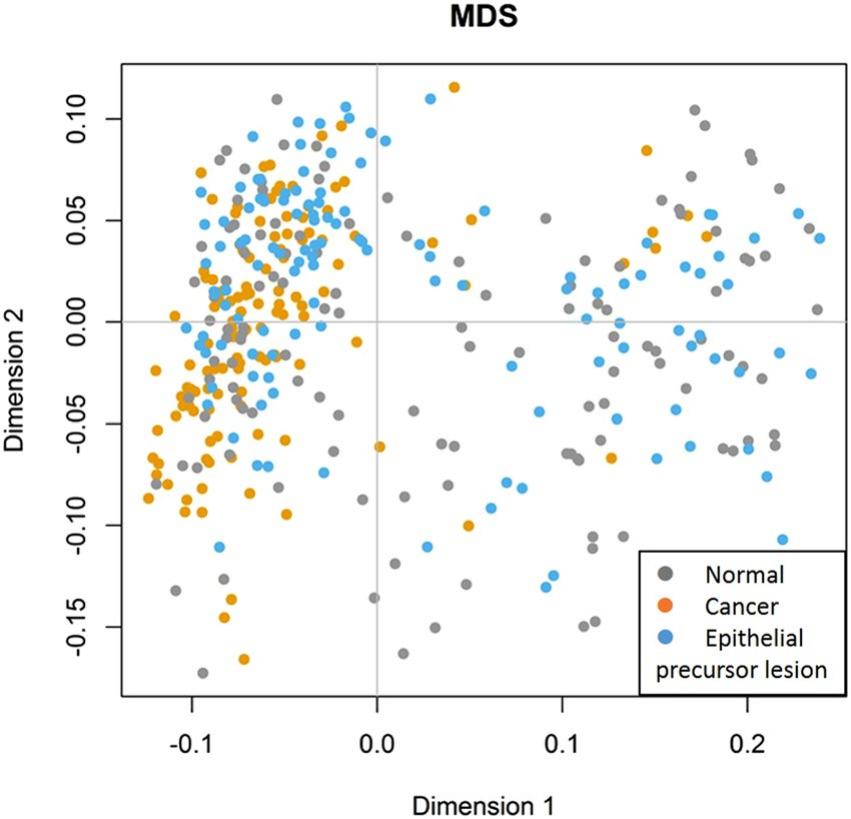
Multidimensional scaling ordination plot of salivary bacterial communities based on the unweighted UniFrac distance metric. Individuals are represented by points. Although the samples did not cluster separately based on groups or subgroups, the distribution in oral cancer patients was more concentrated than that in healthy controls or patients with epithelial precursor lesions.

To assess the diversity among groups, we evaluated the UniFrac distance metric, which measures the evolutionary distance between microbiotas. Due to the sample size, *P* values were measured using the Wilcoxon rank-sum test. Most conditions revealed statistically significant differences between any two subgroups (Fig. 2, Table S7), particularly for former chewers and current smokers. Using the multidimensional scaling method for UniFrac phylogenetic distances, we also found that the samples did not cluster separately into groups or subgroups. Instead, unweighted UniFrac distance analysis revealed that the distribution was more concentrated in the Normal group than in patients with OSCC or epithelial precursor lesions (Fig. 3). According to multi-response permutation procedures (MRPPs), the differences between groups were small, even if statistically significant (*P* < 0.001). We also found statistically significant (*P* < 0.001) differences between subgroups classified according to betel quid chewing status but not with respect to cigarette smoking status.

**Figure 4:**
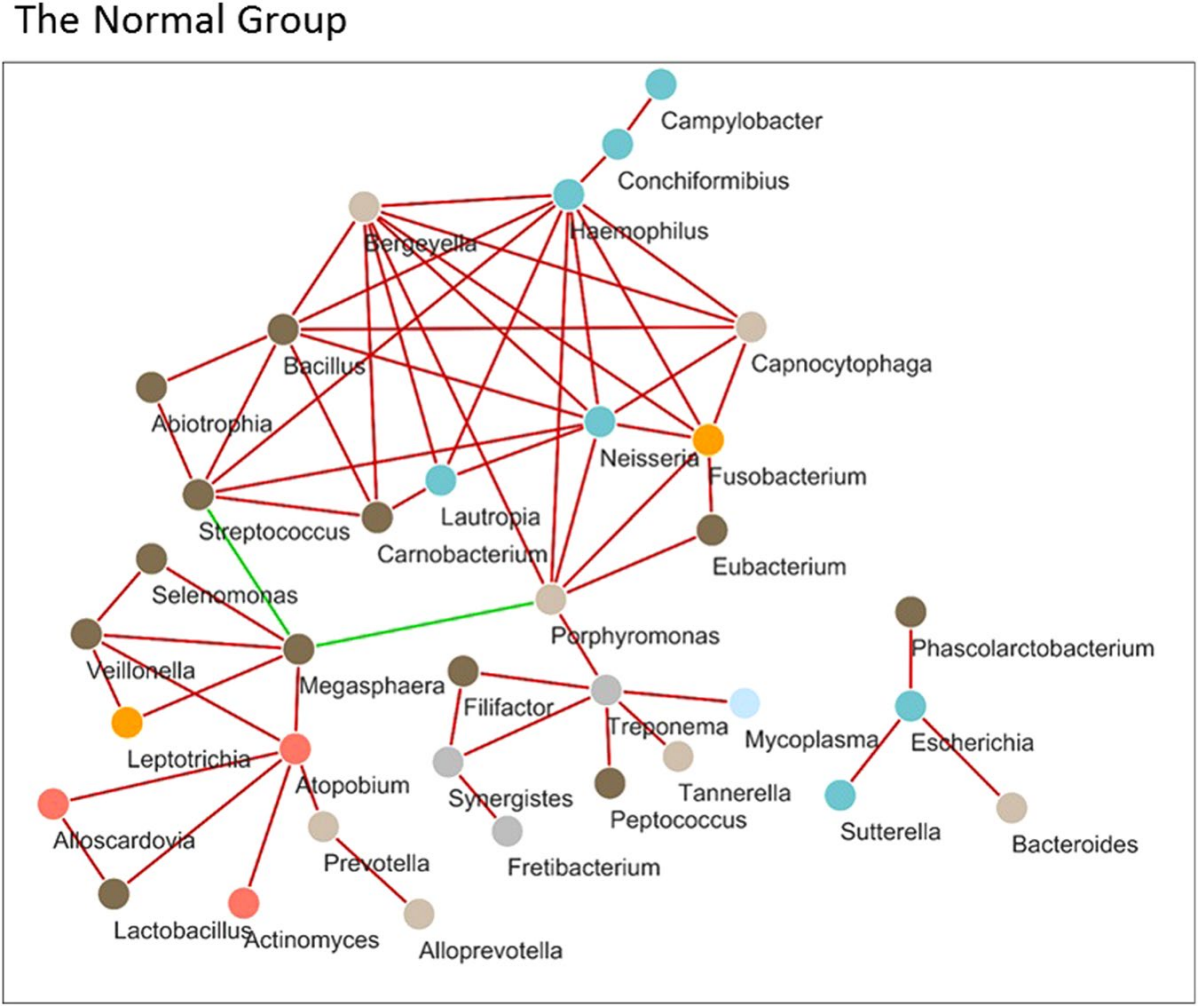
Network analysis of salivary microbiota using SparCC correlation coefficients (Normal group). The figure shows networks between abundant sequences at the genus level built from SparCC correlation coefficients. The nodes represent genera of bacteria; the edges represent the correlation coefficients between genera. The edges are coloured green for negative correlations and red for positive correlations. Nodes of networks are shown when their correlation coefficients are in the top 60 absolute values of the correlation coefficients. In this figure, the number of nodes is 35, and the range of the absoulte values of the correlation coefficients is from 0.339 to 0.622. Nodes are coloured according to their phylum.

**Figure 5:**
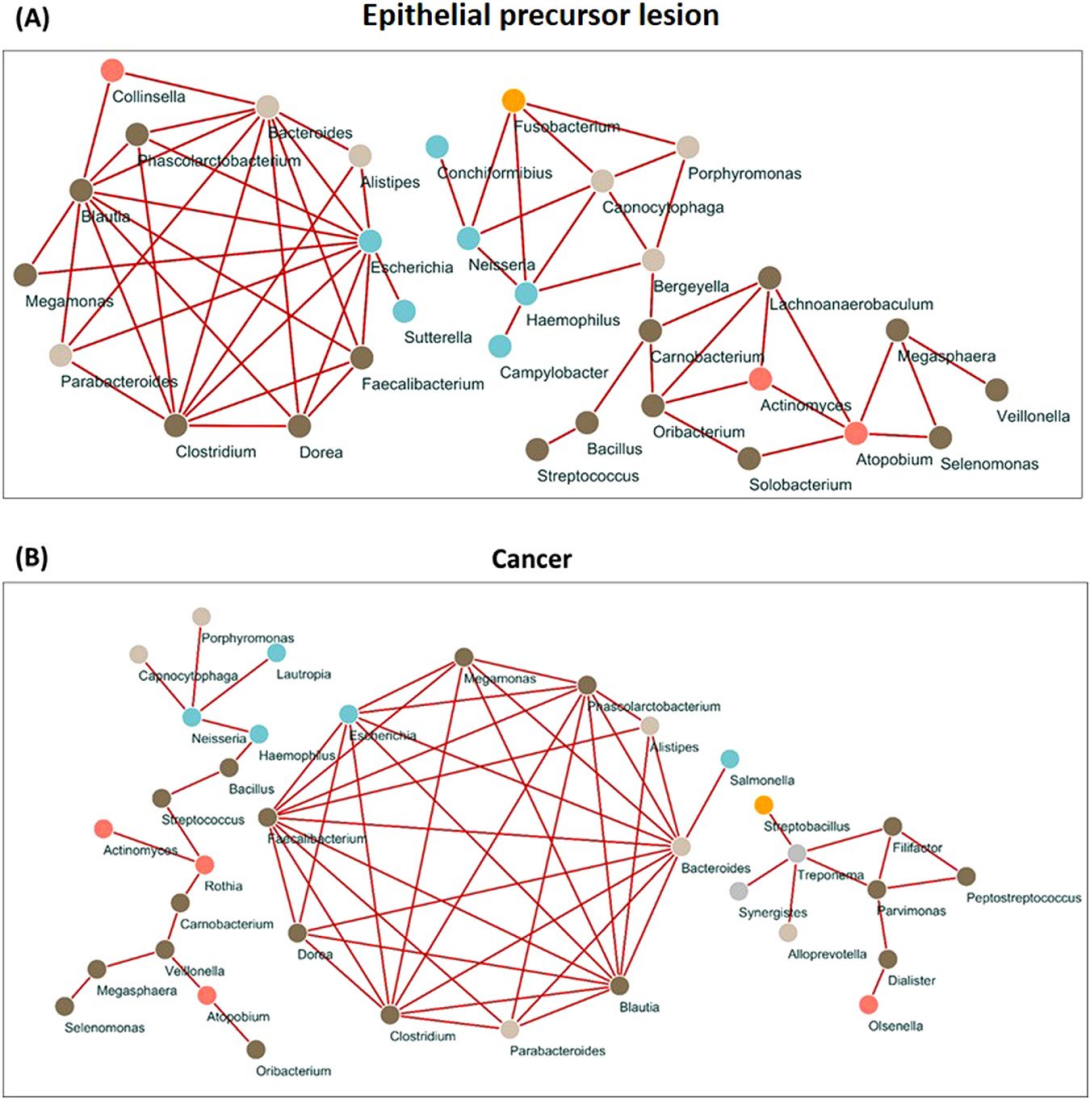
Network analysis of salivary microbiota using SparCC correlation coefficients [Epithelial precursor lesion (**A**) and Cancer groups (**B**)]. (**A**) The number of nodes is 31, and the absolute values of the correlation coefficient range from 0.464 to 0.753. Indeed, these correlations are all positive. (**B**) The number of nodes is 35, and their correlation coefficients range from 0.428 to 0.701. In general, these two networks seem to be similar, but the genus *Parvimonas* is only present in the cancer network.

The number of genera identified in the study was 200. We first selected 83 genera considering that a genus should be present in at least 21% of all individuals. Then, a network was built using SparCC correlation coefficients. All nodes of the networks with coefficients in the top 60 absolute values of SparCC correlation coefficients for each population are shown (Figs 4 and 5). Their ranges were 0.339–0.622, 0.464–0.753, and 0.428–0.701 in the Normal, Epithelial precursor lesion, and Cancer groups, respectively. Finally, 35, 31, and 35 genera were identified in the networks of the Normal, Epithelial precursor lesion, and Cancer groups, respectively. To aid interpretation, nodes are coloured according to their phylum. Interestingly, *Megasphaera* correlated negatively with both *Porphyromonas* and *Streptococcus* among healthy controls; however, in the remaining groups, the correlation coefficients’ top 60 absolute values were all positive. Furthermore, nine bacteria, *Alistipes*, *Bacteroides*, *Blautia*, *Clostridium*, *Dorea*, *Escherichia*, *Faecalibacterium*, *Megamonas*, and *Phascolarctobacterium*, displayed positive correlations with each other in the Epithelial precursor lesion and Cancer groups.

**Figure 6:**
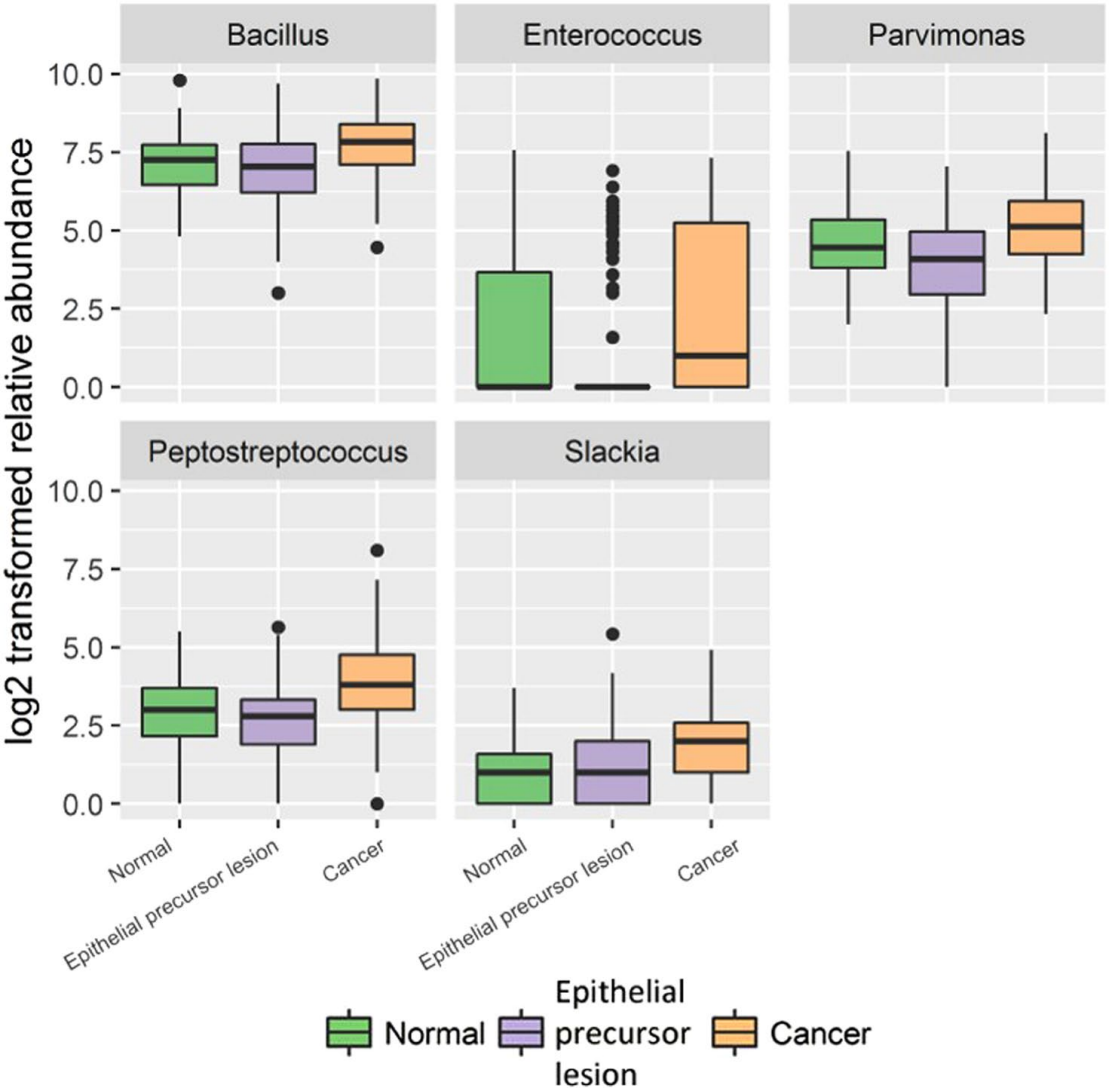
Boxplots of relative abundance levels for five genera among the Normal, Epithelial precursor lesion, and Cancer Populations. The five genera *Bacillus*, *Enterococcus*, *Parvimonas*, *Peptostreptococcus*, and *Slackia* revealed significant differences between the Epithelial precursor lesion and Cancer populations. Except for *Enterococcus*, the others are present in almost all samples.

**Figure 7:**
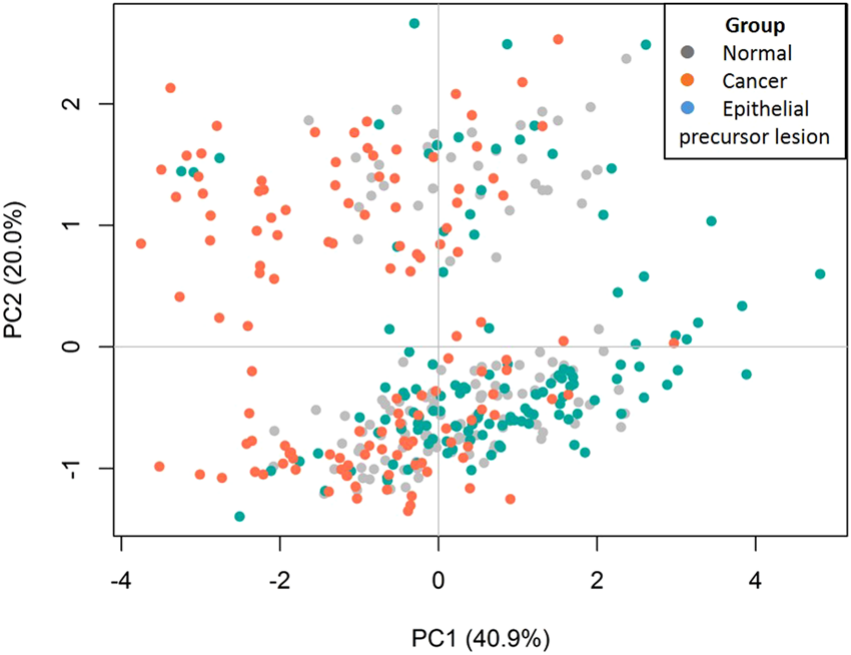
Principal component analysis for five genera: *Bacillus*, *Enterococcus*, *Parvimonas*, *Peptostreptococcus* and *Slackia*. The five genera *Bacillus*, *Enterococcus*, *Parvimonas*, *Peptostreptococcus*, and *Slackia* revealed significant differences between the Epithelial precursor lesion and Cancer populations. These genera seem to roughly classify patients into two clusters. Points represent individuals.

Next, we compared *P* values for 83 genera of bacteria between the patient groups based on cigarette smoking and betel quid chewing status. Given that the ages of non-smokers and non-chewers ranged widely among the healthy controls and that their minimum age among OSCC patients was 55 years, we limited the age of healthy controls to 55 years or older (Additional File 1). Accordingly, in the B−C− subgroup, seven genera, *Filifactor*, *Fretibacterium*, *Lachnoanaerobaculum*, *Megasphaera*, *Mycoplasma*, *Peptostreptococcus*, and *Tannerella*, were present in nearly all samples, with statistically significant differences between the Normal and Cancer groups. Ten genera revealed significant differences between the Normal and Cancer groups (Figs S12 and S13). Regarding the Epithelial precursor lesion and Normal groups, patients with epithelial precursor lesions could be classified into two parts by 31 genera (Fig. S14, Additional File 2). We also found that five genera, *Bacillus*, *Enterococcus*, *Parvimonas*, *Peptostreptococcus*, and *Slackia*, exhibited significant differences between the Epithelial precursor lesion and Cancer groups, roughly classifying these patients into two clusters (Figs 6 and 7, S15, and Additional File 3).

**Figure 8:**
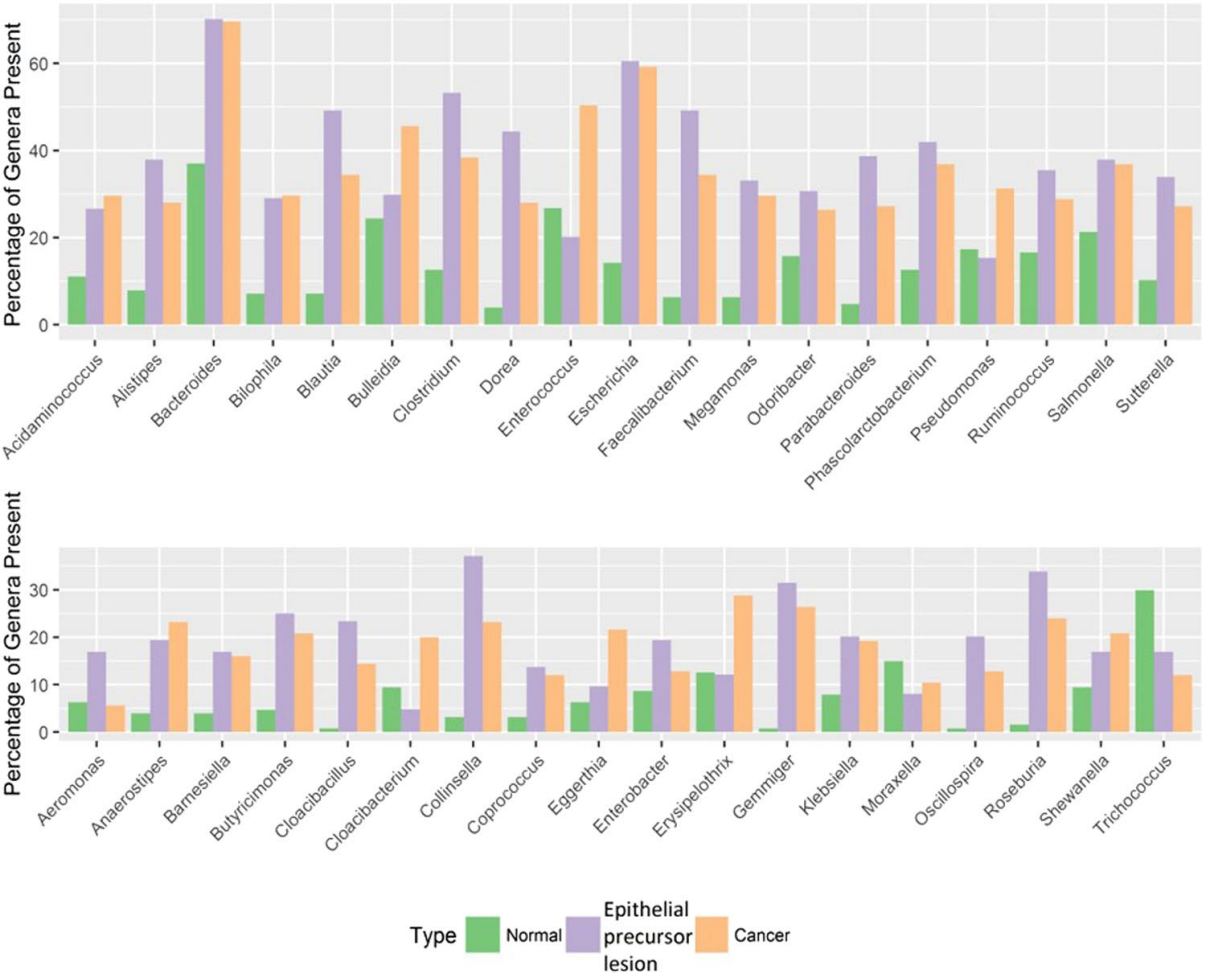
The percentage of selected genera present in each group. Thirty-seven genera that met the following conditions were selected: (1) the genus was present in 11–59% of samples in a group and (2) the genus was more than twice as abundant in that group as in any other group.

We further examined 37 genera of bacteria that met the following conditions: 1) the genus was present in 11–59% of samples from one patient group, and 2) the genus was more than twice as abundant in that group than in any other group (Fig. 8). We found that *Bacteroides* and *Escherichia* were present at over 60% in the Epithelial precursor lesion and Cancer groups but at only 37% and 14.2%, respectively, in the Normal group. In addition, the percentages of *Bulleidia* were similar in the Normal and Epithelial precursor lesion groups, whereas it was approximately 46% in the Cancer group. Furthermore, the four genera *Blautia*, *Dorea*, *Faecalibacterium*, and *Phascolarctobacterium* were present in 49.2%, 44.4%, 49.2%, and 41.9%, respectively, of the Epithelial precursor lesion subjects; however, they were present in less than 13% of the Normal subjects. Indeed, these four genera displayed strong positive correlations with respect to network analysis (Fig. 8). Notably, *Cloacibacillus*, *Gemmiger*, *Oscillospira*, and *Roseburia* were 20 times more abundant in Epithelial precursor lesion and Cancer patients than in Normal subjects, in whom the sum of the relative abundance for each one was less than three. In addition, other poorly abundant genera might also be associated with epithelial precursor lesions or OSCC.

## Discussion

The purpose of the present investigation was to compare the unstimulated salivary microbial profiles of patients with epithelial precursor lesions, patients with oral cancer, and normal controls. The results from this investigation demonstrated that correlation networks of salivary microbiota were remarkably varied within the three groups and that five genera – *Bacillus*, *Enterococcus*, *Parvimonas*, *Peptostreptococcus*, and *Slackia* – indicated significant differences between the Epithelial precursor lesion and Cancer groups.

In our study, most oral cancer patients were former or current chewers, as were patients with epithelial precursor lesions. This finding is consistent with early reports that betel quid chewing is a major causal agent of oral cancer^3,40^. That is, a small proportion of individuals were non-chewers and non-smokers within the Epithelial precursor lesion and Cancer groups. The number of both non-chewers and non-smokers was 14 in the Epithelial precursor lesion group, of whom 8 were female and 6 were male. However, in the Cancer group, 7 of 9 patients who were non-smokers and non-chewers were female, and these females were all non-drinkers. This result is in accord with recent research reporting that non-chewers and non-smokers among oral cancer patients tends towards a higher proportion of females, but the cause of this phenomenon is still unclear^41^.

Our high-throughput NGS platform successfully identified more than 500 species, including cultured and uncultured strains, using saliva, which is a source with low sensitivity. This large dataset represents a significant improvement compared with the approximately 50 strains detected using traditional experimental methods. Due to the nature of oral neoplasms, the diagnosis of an epithelial precursor lesion is relatively easy compared with that of other types of cancer. However, the transition from epithelial precursor lesion to cancer is slow and requires further and continuous follow-up. Microbial community changes (*Bacillus*, *Enterococcus*, *Parvimonas*, *Peptostreptococcus*, and *Slackia*) in the saliva might represent a convenient marker for the prediction, detection, and prognosis of oral cancer, especially the epithelial precursor lesion-cancer transition. Furthermore, in the genus-level sequence abundance network analysis from SparCC correlation coefficients, we identified at least 9 bacteria – *Alistipes*, *Bacteroides*, *Blautia*, *Clostridium*, *Dorea*, *Escherichia*, *Faecalibacterium*, *Megamonas*, and *Phascolarctobacterium* – that displayed positive correlations with each other in the Epithelial precursor lesion and Cancer groups. Additionally, a very different positive/negative correlation network was detected in the Normal group, suggesting the presence of a community alteration with disease. These correlations may suggest that the microbial community in saliva changes in a group manner and that we should study these alterations in groups, which could result in the identification of various combinations of bacteria correlated with oral cancer and epithelial precursor lesions. In conclusion, we systematically investigated microbiota changes between normal, epithelial precursor lesion, and oral cancer patients and report that five genera revealed significant differences between the Epithelial precursor lesion and Cancer groups. The present study demonstrates that changes in microbiota composition might have the potential to be used as a biomarker for predicting the pathologic development transition of oral epithelial precursor lesion to cancer. Oral health conditions were not included in this study since large numbers of oral cancer and epithelial precursor lesion patients in Taiwan are betel quid consumers. The habit of regular betel chewing usually causes health problems in the oral cavity, such as periodontitis. Therefore, even the normal controls recruited in this study had a certain percentage of betel quid consumers, which actually reflects the reality of the composition of patients visiting oral and dental medical centres in Taiwan’s hospitals. We designed this study in an attempt to develop a tool to help physicians of practical medicine who encounter patients with different life habits. We will try to collect more healthy controls and patients without the habits of betel chewing or smoking for more precisely defined healthy controls to eliminate the influences of betel chewing and smoking. Future studies should include larger numbers of samples collected prospectively from multiple hospitals and should investigate the association between microbiota changes and the clinicopathological characteristics of oral cancer.

## Methods

### Ethics approval and consent to participate

The study was reviewed and approved by the Ethics Committee of Chung Shan University Hospital (CSMUH No: CS13214). All of the methods were performed in accordance with relevant guidelines and regulations, including any relevant details. Informed consent was obtained from the patients and was approved by the Institutional Review Board.

### Patients and sample collection

The aim of the study was to identify possible correlations between lifestyle habits and microbiome composition in individuals with OSCC. All participants were patients from Chung Shan Medical University Hospital, Taichung, Taiwan between 2014 and 2015. Overall, the dataset comprised 376 human saliva samples, including 125 cases of OSCC; 124 cases of epithelial precursor lesion with histopathological evidence of dysplasia, hyperplasia, or hyperkeratosis; and 127 normal controls with no malignant disease in the oral cavity. Patients with only primary untreated OSCC, including chemotherapy or radiotherapy, were recruited. Patients and normal controls with any history of diabetes mellitus or immune system-related disease were excluded. All individuals were free of antibiotic therapy for three months prior to the study. Saliva samples (5 mL) from each participant were collected unstimulated, at least 1 h after eating, drinking, or smoking; or after mouth rinsing and waiting for 10 min before saliva collection if the patient ate within one hour^42^. Saliva samples were collected in test tubes and stored at −80 °C. The patients completed a questionnaire to disclose information regarding sex, age, alcohol consumption, betel chewing, cigarette smoking, and family history of cancer.

### DNA extraction

DNA was extracted directly from saliva using the QIAamp DNA Blood Mini kit (Qiagen, USA). Each sample was transferred to a 1.5-mL microcentrifuge tube and centrifuged at 18,000 × *g* for 2 min to pellet the bacteria. The pellets were re-suspended and then treated with lysozyme in enzyme solution (20 mM Tris-HCl, pH 8.0; 2 mM EDTA; 2% SDS) at 37 °C and proteinase K in buffer AL at 56 °C and 95 °C. Extraction was performed following the manufacturer’s protocol using a QIAamp spin column. The DNA was eluted with 50 μL of Buffer AE. All samples were centrifuged at 18,000 × *g* for 1 min, the DNA concentration was measured with a NanoPhotometer® (Implen, Germany), and the samples were stored at −20 °C until further analysis.

### Library construction and sequencing of the 16S ribosomal DNA (rDNA) V4 region

The PCR primer pair F515 (5′-GTGCCAGCMGCCGCGGTAA-3′) and R806 (5′-GGACTACHVGGGTWTCTAAT-3′) was designed to amplify the V4 region of bacterial 16S rDNA as previously described^43^. PCR amplification was performed in a 50-μL reaction volume containing 25 μL of 2× Taq Master Mix (Thermo Scientific, USA), 0.2 μM of each primer, and 20 ng of DNA template. The reaction process was performed at 95 °C for 5 min, followed by 30 cycles at 95 °C for 30 sec, 54 °C for 1 min, and 72 °C for 1 min, plus a final extension at 72 °C for 5 min. Subsequently, the amplified products were checked by 2% agarose gel electrophoresis and ethidium bromide staining. Amplicons were purified using an AMPure XP PCR Purification Kit (Agencourt, Beckman Coulter, USA) and were quantified using the Qubit dsDNA HS Assay Kit (Life Technologies, USA) with a Qubit 2.0 fluorometer following the respective manufacturers’ instructions.

For V4 library preparation, Illumina adapters were attached to the amplicons using the Illumina TruSeq DNA Sample Preparation v2 Kit (Illumina, USA). Purified libraries were processed for cluster generation and sequencing using the MiSeq system.

### Filtering 16S rRNA sequencing data for quality

Sequencing reads from different samples were identified and separated using specific barcodes at the 5′ end of the sequence (two mismatches allowed). The FASTX-Toolkit (http://hannonlab.cshl.edu/fastx_toolkit) was employed to process raw read data files. We performed three steps to ensure sequence quality processing. (i) The command “fastq_quality_filter −Q33 −q 20 −p 70”. “−q 20” denoted that the minimum quality score was 20. “−p 70” defined the minimum percentage of bases required for “−q” quality to be over or equal to 70%. (ii) The command “fastq_quality_trimmer −t 20 −l 100 −Q33”. “−t 20” denoted that bases with lower quality (<20) would be trimmed (checking from the end of the sequence). “−l 100” indicated that the minimum acceptable sequence length was 100 after trimming. (iii) Sequences were retained if both forward and reverse sequencing reads passed the first and second steps.

### Taxonomy assignment based on bacterial 16S rRNA sequences

To assign taxonomy, a collection of 16 S rRNA sequences was retrieved from the SILVA ribosomal RNA sequence database (release 115)^44,45^. These sequences were extracted with V4 forward and reverse primers. Then, UCLUST was used to create representative sequence clusters with similarities equalling or above 97%^46^. Bowtie2^47^ was used to align sequencing reads against V4 sequence clusters. A 97% similarity standard was applied to V4 sequence clusters.

### Statistical analysis

The OTU table of raw counts was normalised to an OTU table of relative abundance values. Taxa of the same type were agglomerated at the phylum, class, order, family, and genus levels via the SILVA database. Biodiversity was compared between classified groups using the nonparametric Wilcoxon test. Kendall’s Tau and Spearman’s rank correlation coefficients were used to test the association between richness (species number) and the Shannon diversity index. To investigate the relationships between bacteria, we first calculated the average sum of relative abundance for each genus in the whole sample and then used principal component analysis for the top 15 genera. Phylogeny-based UniFrac analysis was also performed^48^. Clustering and multidimensional scaling (also known as principal coordinate analysis) methods were used for visualisation based on data reduction of patterns in an n-dimensional dataset. MRPPs were used to test differences between groups. Based on the characteristics of the compositional data, a network of specific genera was built using SparCC correlation coefficients^49^. R software was used for statistical analysis (The R Project for Statistical Computing, Vienna, Austria).

### Availability of data and materials

Sequence data associated with this project have been deposited at the NCBI under study accession SRP107079.

## Electronic supplementary material

Supplementary Information

Dataset 1

Dataset 2

Dataset 3
