# Supplementary material for: Bacterial alterations in salivary microbiota and their association in oral cancer
Source: Sci Rep. 2017 Nov 28;7:16540. doi: 10.1038/s41598-017-16418-x (PMC5705712; doi:10.1038/s41598-017-16418-x)
Supplement: Supplementary file 1 — Supplementary Information [file 41598_2017_16418_MOESM1_ESM.pdf]

Supplementary Information

Bacterial alterations in salivary microbiota and their association in oral cancer

Wei-Hsiang Lee[1,2,3†], Hui-Mei Chen[3†], Shun-Fa Yang[1,4†], Chao Liang[3], Chih-Yu Peng[5,6], Feng-Mao Lin[3], Lo-Lin Tsai[5,6], Buor-Chang Wu[5,6], Chung-Han Hsin[7,8], Chun-Yi Chuang[7,8], Ting Yang[3], Tzu-Ling Yang[3], Shinn-Ying Ho[3], Wen-Liang Chen[2], Kwo-Chang Ueng[1], Hsien-Da Huang[2,3*], Chien-Ning Huang[4,9*], Yuh-Jyh Jong[2,3,10,11*]

[1]Department of Medical Research, Chung Shan Medical University Hospital, Taichung 402, Taiwan

[2]Department of Biological Science and Technology, National Chiao Tung University, Hsinchu 300, Taiwan

[3]Institute of Bioinformatics and Systems Biology, National Chiao Tung University, Hsinchu 300, Taiwan

[4]Institute of Medicine, Chung Shan Medical University, Taichung 402, Taiwan

[5]School of Dentistry, Chung Shan Medical University, Taichung 402, Taiwan

[6]Department of Dentistry, Chung Shan Medical University Hospital, Taichung 402, Taiwan

[7]School of Medicine, Chung Shan Medical University, Taichung 402, Taiwan

[8]Department of Otolaryngology, Chung Shan Medical University Hospital, Taichung 402, Taiwan

[9]Department of Internal Medicine, Division of Endocrinology and Metabolism, Chung Shan Medical University Hospital, Taichung 402, Taiwan

[10]Graduate Institute of Clinical Medicine, College of Medicine, Kaohsiung Medical University, Kaohsiung 807, Taiwan

[11]Departments of Paediatrics and Laboratory Medicine, Kaohsiung Medical University Hospital, Kaohsiung Medical University, Kaohsiung 807, Taiwan

[*]Correspondence: bryan@mail.nctu.edu.tw; cshy049@gmail.com; yjjongnctu@gmail.com

[†] These authors contributed equally to this work

Table S1. Descriptive statistics for age among subgroups based on the status of betel nut chewing and smoking cigarettes

Subgroup	Group	Number of person	min	mean	max	SD	SE
B−C− (n=85)	N	62	24	53.1	90	15.6	2.0
	P	14	41	55.6	76	10.7	2.9
	C	9	55	67.4	80	10.1	3.4
B−C* (n=25)	N	14	36	57.6	89	14.6	3.9
	P	10	45	55.1	67	7.5	2.4
	C	1	43	43.0	43	NA	NA
B−C+ (n=36)	N	16	30	47.1	56	7.6	1.9
	P	17	35	49.3	68	9.4	2.3
	C	3	43	53.0	64	10.5	6.1
B*C− (n=14)	N	0	NA	NA	NA	NA	NA
	P	4	44	48.5	56	5.4	2.7
	C	10	40	56.8	74	10.4	3.3
B*C* (n=63)	N	11	43	60.4	83	14.2	4.3
	P	16	31	56.9	80	14.4	3.6
	C	36	41	56.7	79	10.4	1.7
B*C+ (n=88)	N	7	35	49.0	62	12.4	4.7
	P	36	25	50.6	70	11.7	1.9
	C	45	31	49.5	68	9.0	1.3
B+C− (n=5)	N	1	41	41.0	41	NA	NA
	P	2	34	42.0	50	11.3	8.0
	C	2	41	46.5	52	7.8	5.5
B+C* (n=5)	N	2	42	45.0	48	4.2	3.0
	P	1	48	48.0	48	NA	NA
	C	2	44	51.5	59	10.6	7.5
B+C+ (n=55)	N	14	31	46.1	66	11.4	3.0
	P	24	23	43.3	71	11.2	2.3
	C	17	34	47.9	62	10.0	2.4

1) N: Normal (Healthy control); P: epithelial Precursor lesion; C: Cancer
2) B−: Never chewer;
B*: Former chewer;
B+: Current chewer;
C−: Never smoker;
C*: Former smoker;
C+: Current smoker
3) SD: Standard deviation; SE: Standard error

Table S2. Percentage of former chewer or current chewer within groups

Sample Groups	*Former chewer or current chewer* Yes	No	Total
Normal<br>% within normal	35<br>28%	92<br>72%	127<br>100%
Epithelial precursor lesion<br>% within epithelial precursor lesion	83<br>67%	41<br>33%	124<br>100%
Cancer<br>% within cancer	112<br>90%	13<br>10%	125<br>100%
Total<br>% within groups	230<br>61%	146<br>39%	376<br>100%

Table S3. Percentage of former smoker or current smoker within groups

Sample Groups	*Former smoker or current smoker* Yes	No	Total
Normal<br>% within normal	64<br>50%	63<br>50%	127<br>100%
Epithelial precursor lesion<br>% within epithelial precursor lesion	104<br>84%	20<br>16%	124<br>100%
Cancer<br>% within cancer	104<br>83%	21<br>17%	125<br>100%
Total<br>% within groups	272<br>72%	104<br>28%	376<br>100%

Table S4. The p-values for UniFrac distance between groups

		Normal vs Epithelial precursor lesion	Normal vs Cancer	Epithelial precursor lesion vs Cancer
B−C−	Unweighted	0.0267	0.0028	0.1321
	Weighted	0.1899	0.8516	0.7140
B−C*	Unweighted	6.09E-14	NA	NA
	Weighted	0.8532	NA	NA
B−C+	Unweighted	0.0506	0.0136	0.0152
	Weighted	0.0010	0.3293	0.3069
B*C*	Unweighted	3.37E-15	8.59E-07	5.08E-08
	Weighted	0.0022	0.0120	0.0806
B*C+	Unweighted	4.94E-06	0.0002	0.0002
	Weighted	0.0070	0.0166	0.0391
B+C+	Unweighted	6.04E-08	0.52063	1.65E-07
	Weighted	0.0049	0.7563	0.0001

Table S5. Cancer subsites and clinical and pathological staging of cancer subjects

**Oral cavity cancer subsites**	
Tongue	41
Mouth floor	6
Lip	10
Buccal	42
Gum (alveolar ridge)	24
Hard plate	3
Retromolar trigone	0
Others	6
**Primary tumor differentiation**	
Well	33
Moderate	64
Poor	15
Undifferentiated	0
No report	2
**Tumor cell differentiation**	
Squamous cell carcinoma	112
Verrucous carcinoma	0
Others	1

* Some subjects have partial of clinical information omitted.

* Several subjects have more than one cancer subsites.

Table S6. Clinical and pathological staging of cancer subjects

Stage	Clinical stage	Pathological stage
I	39	36
II	13	19
III	12	3
IVA/IVB	51	29
**Tumor status**		
T1	42	43
T2	28	21
T3	4	2
T4a/T4b	41	21
**Lymph node status**		
N0	70	57
N1	17	3
N2b/N2c	27	15
Nx	1	14
**Metastasis status**		
M0	114	88
M1	1	0

* Some subjects have partial of clinical information omitted.

Supplementary Figures

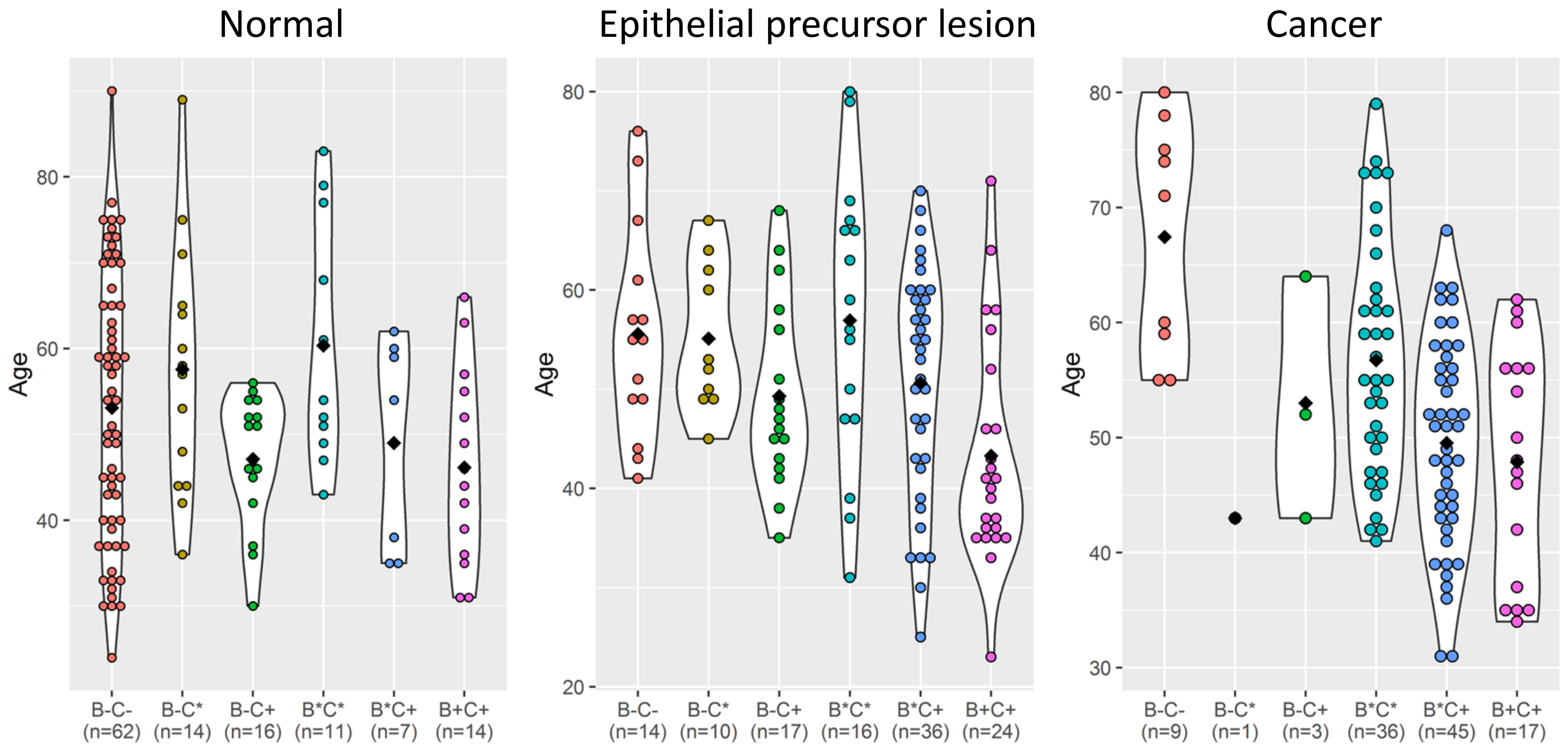

Figure S1. Boxplots of age and groups. The figure shows the distribution by age, represented vertically, and groups, horizontally. Most patients were former or current smokers/chewers. The symbol B− was used to mean non-chewer; the symbol B* was former chewer; the symbol B+ was current chewer. Similarly, the symbol C− was used to mean non-smoker; the symbol C* was former smoker; the symbol C+ was current smoker. The median age of both non-smokers and non-chewers (that is, in the B–C– subgroup) in the Cancer group was much higher than that in the other subgroups.

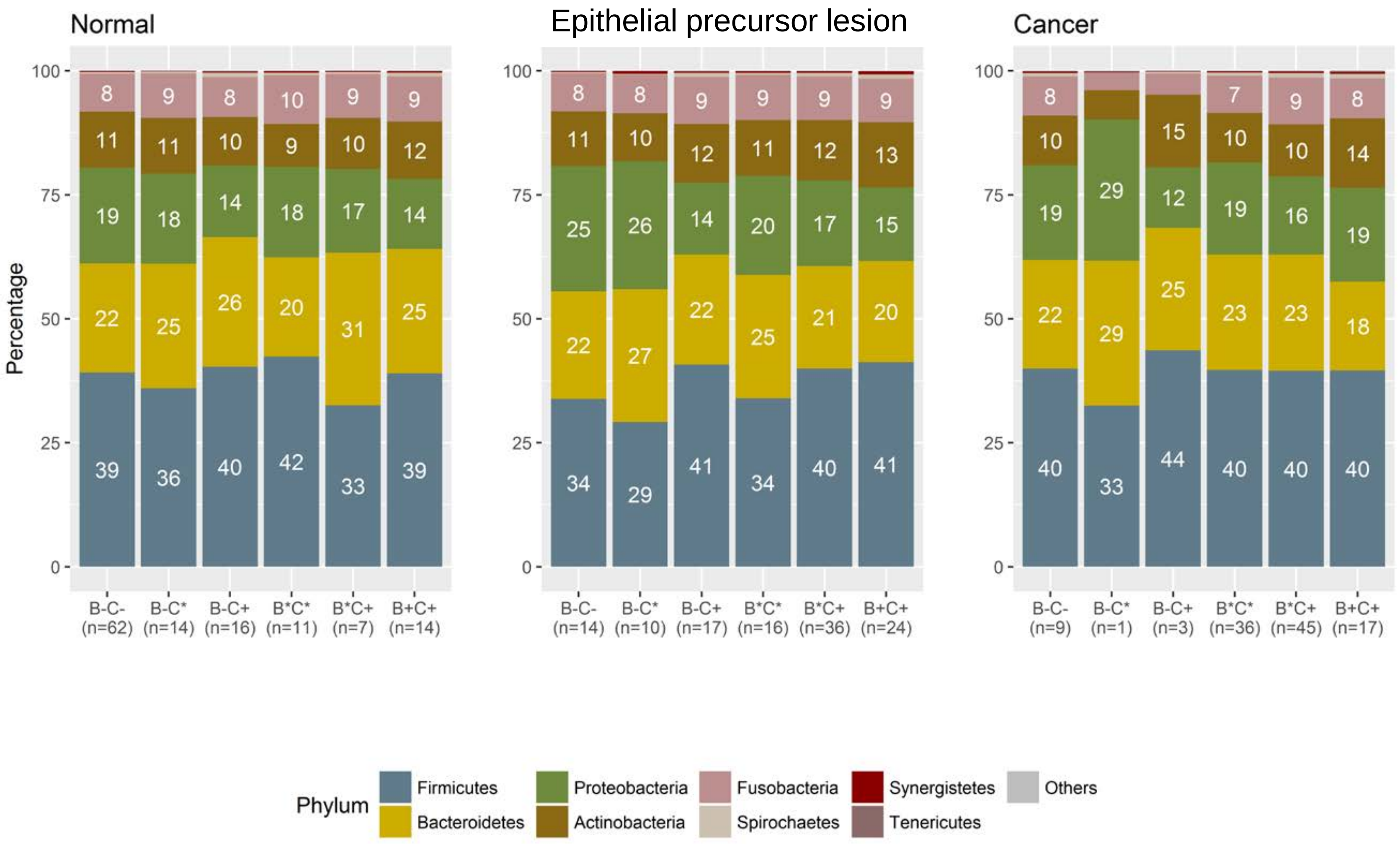

Figure S2. Phylum-level microbial composition of salivary community (legend on the bottom). Firmicutes was the most dominant phyla within each subgroup. The relative abundance of of Firmicutes, Bacteroidetes, and Proteobacteria is similar in the B–C* subgroup of Epithelial precursor lesion patients.

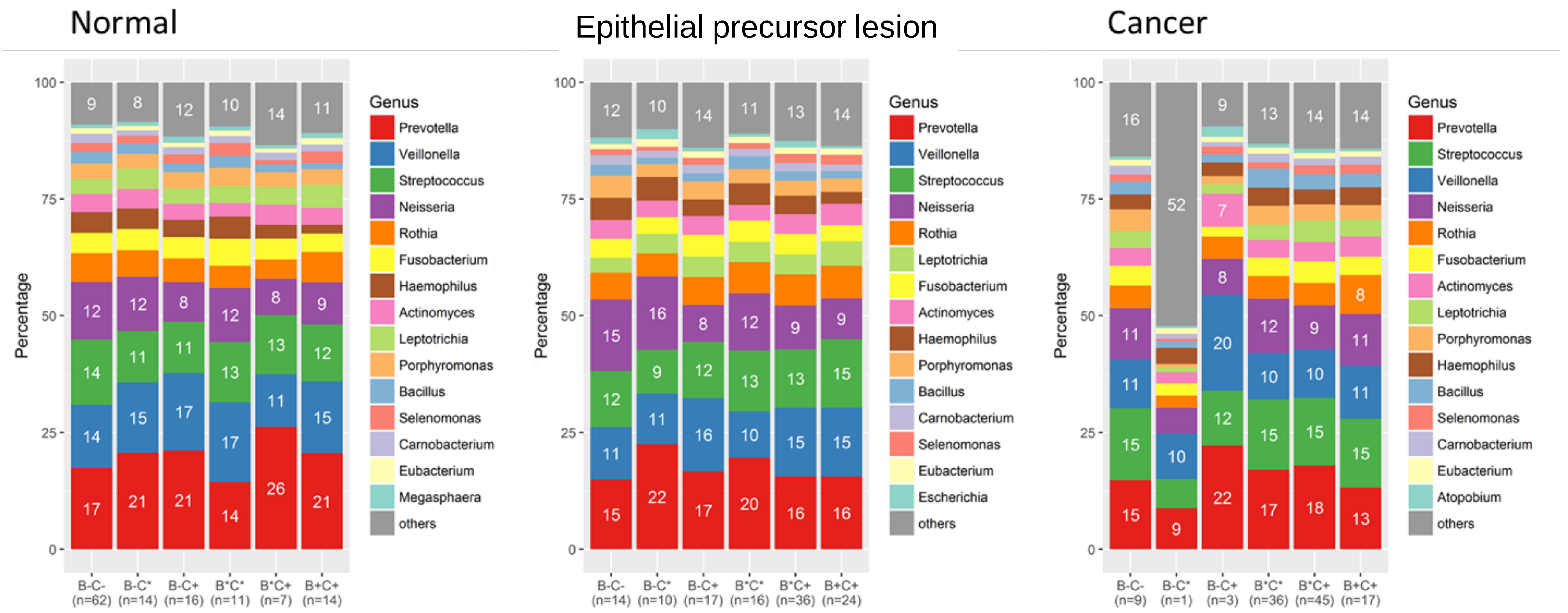

Figure S3. Genus-level microbial composition of the salivary community (legend on the right). The salivary microbiota was evaluated using operational taxonomic units defined by a 97% sequences similarity cutoff. Each color represents one bacterial genus. The three groups shared the same top 14 genera, arranged in just slightly different order. In the Normal group, the relative abundance of *Prevotella* within the B*C* subgroup was less than in the other subgroups, while the relative abundance of *Prevotella* within the B*C+ subgroup was higher than the other ones.

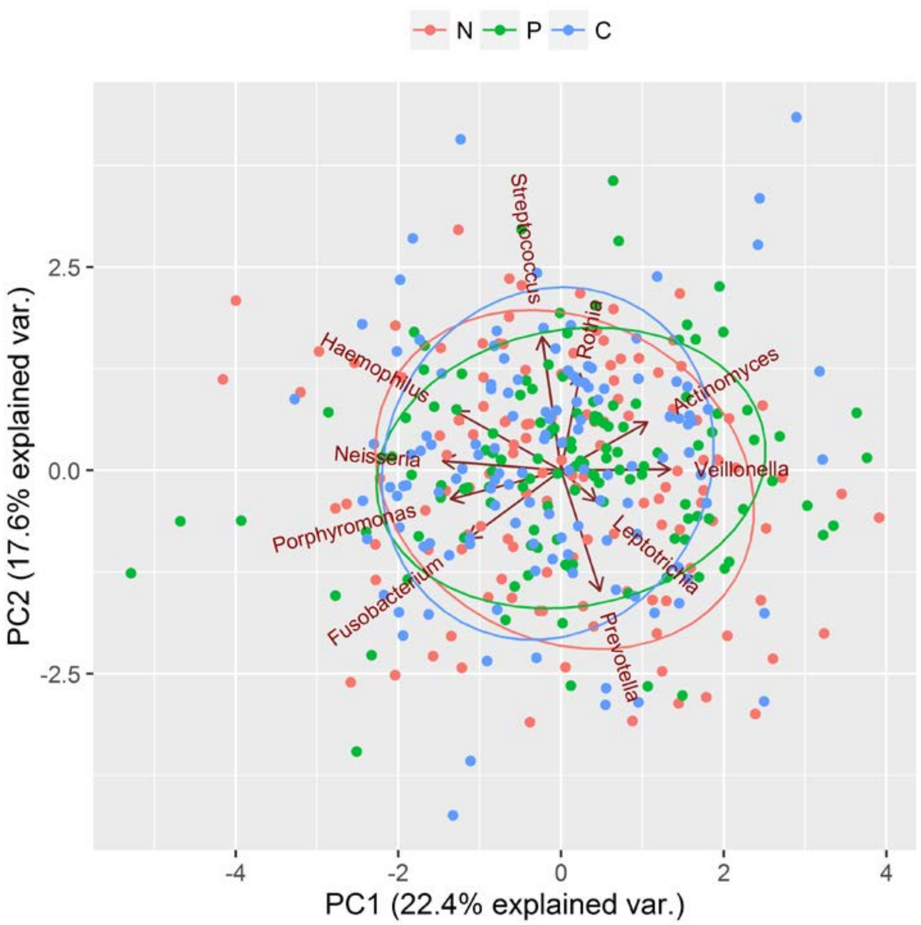

Figure S4. Principal component analysis of (log2-transformed) the top 10 genera of the Normal, Epithelial precursor lesion and Cancer groups. The two first components (PC1 and PC2) were plotted. Points represent individuals. The length of the brown vectors represents the effect of each component on the status. The distance between them indicated their correlation. Circles represent three clusters formed because of close correlations. There was no separately clustering among groups. The letter N was used to mean Normal (pink color); the letter P was used to mean epithelial Precursor lesion (green color); the letter C was used to mean Cancer (blue color).

The Normal group: non-chewers

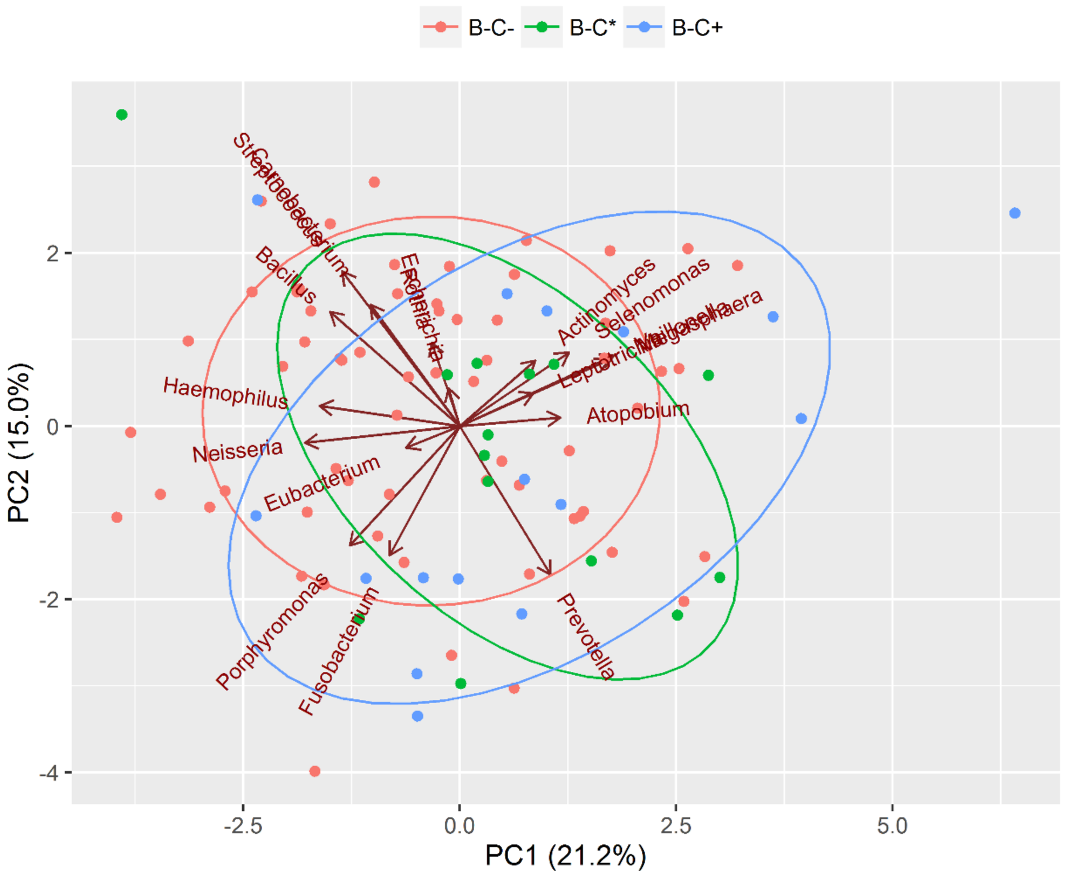

Figure S5. Principal component analysis of (log2-transformed) the top 17 genera for non-chewers in the Normal group. The two first components (PC1 and PC2) were plotted. Points represent individuals. The length of the brown vectors represents the effect of each component on the status. The distance between them indicated their correlation. Circles represent three clusters formed because of close correlations. There was no separately clustering among subgroups. The correlation between *Prevotella* and the other genera was almost negative.

The Epithelial precursor lesion group: non-chewers

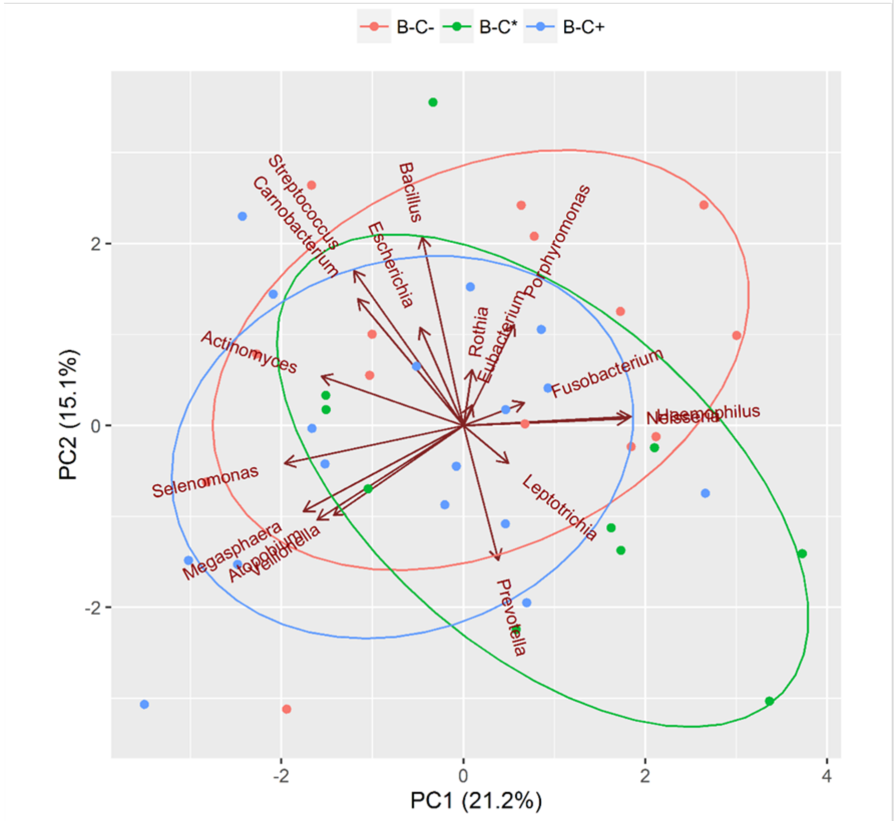

The Cancer group: non-chewers

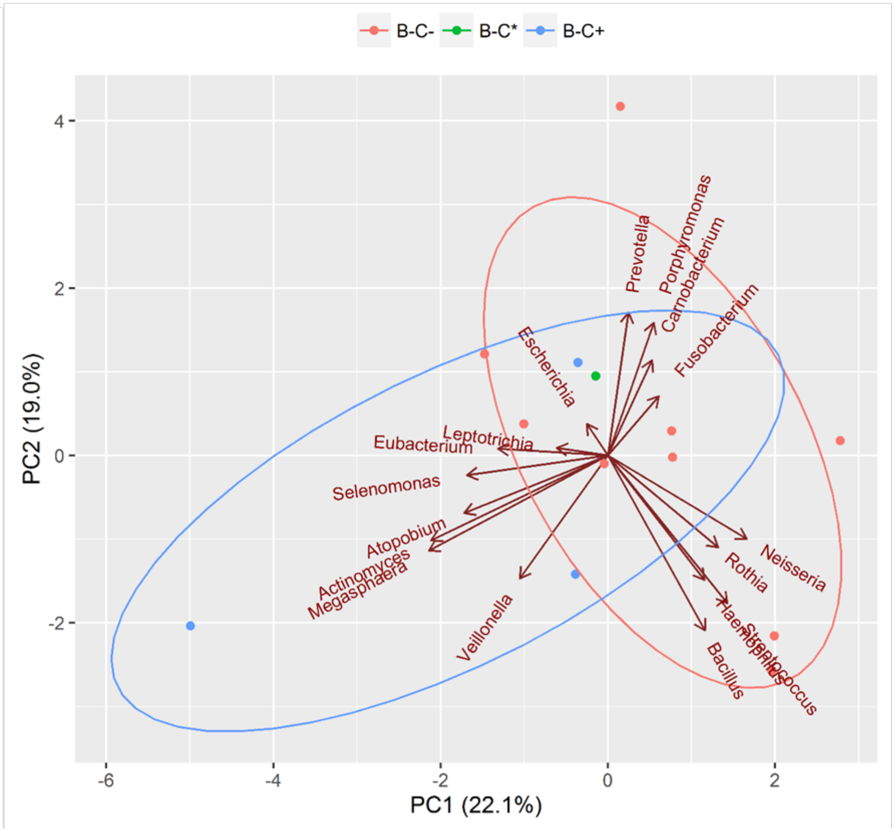

Figure S6. Principal component analysis of (log2-transformed) the top 17 genera for non-chewers in the Epithelial precursor lesion and Cancer groups. The two first components (PC1 and PC2) were plotted. Points represent individuals. The length of the brown vectors represents the effect of each component on the status. The distance between them indicated their correlation. Circles represent three clusters formed because of close correlations. There was no separately clustering among subgroups.

Both current smokers and current chewers

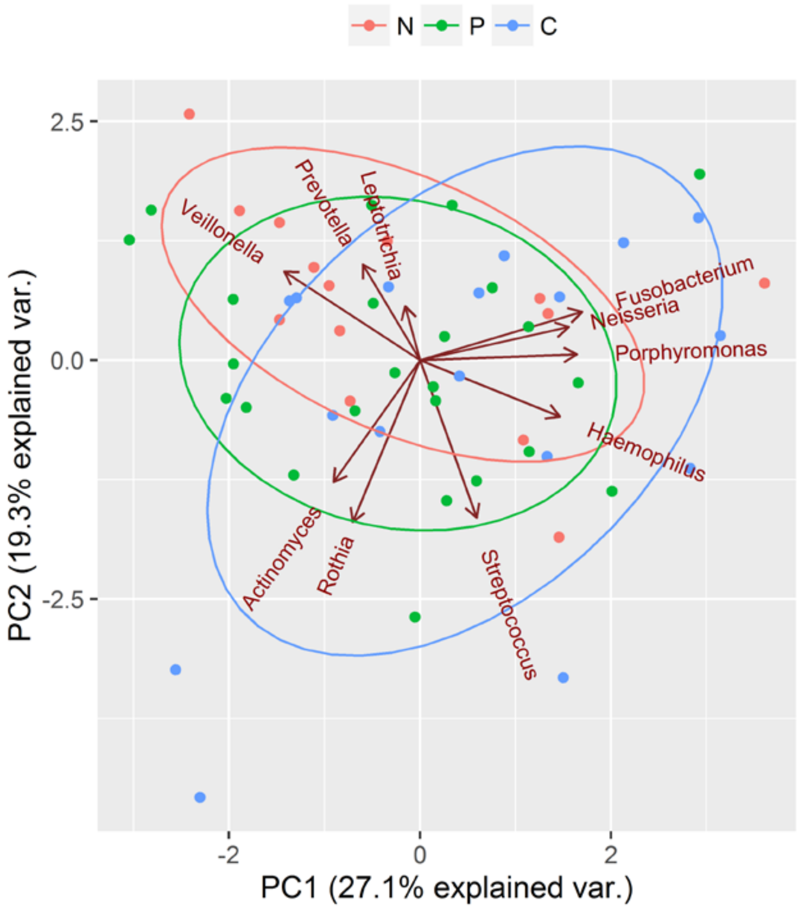

Figure S7. Principal component analysis of (log2-transformed) the top 10 average relative abundances for current smokers and current chewers. The two first components (PC1 and PC2) were plotted. Points represent individuals. The length of the brown vectors represents the effect of each component on the status. The distance between them indicated their correlation. Circles represent three clusters formed because of close correlations. A strong positive correlation was evident between *Prevotella* and *Veillonella* or *Prevotella* and *Leptotrichia* within both current smokers and current chewers.

The Epithelial precursor lesion group: former or current chewers

The Cancer group: former or current chewers

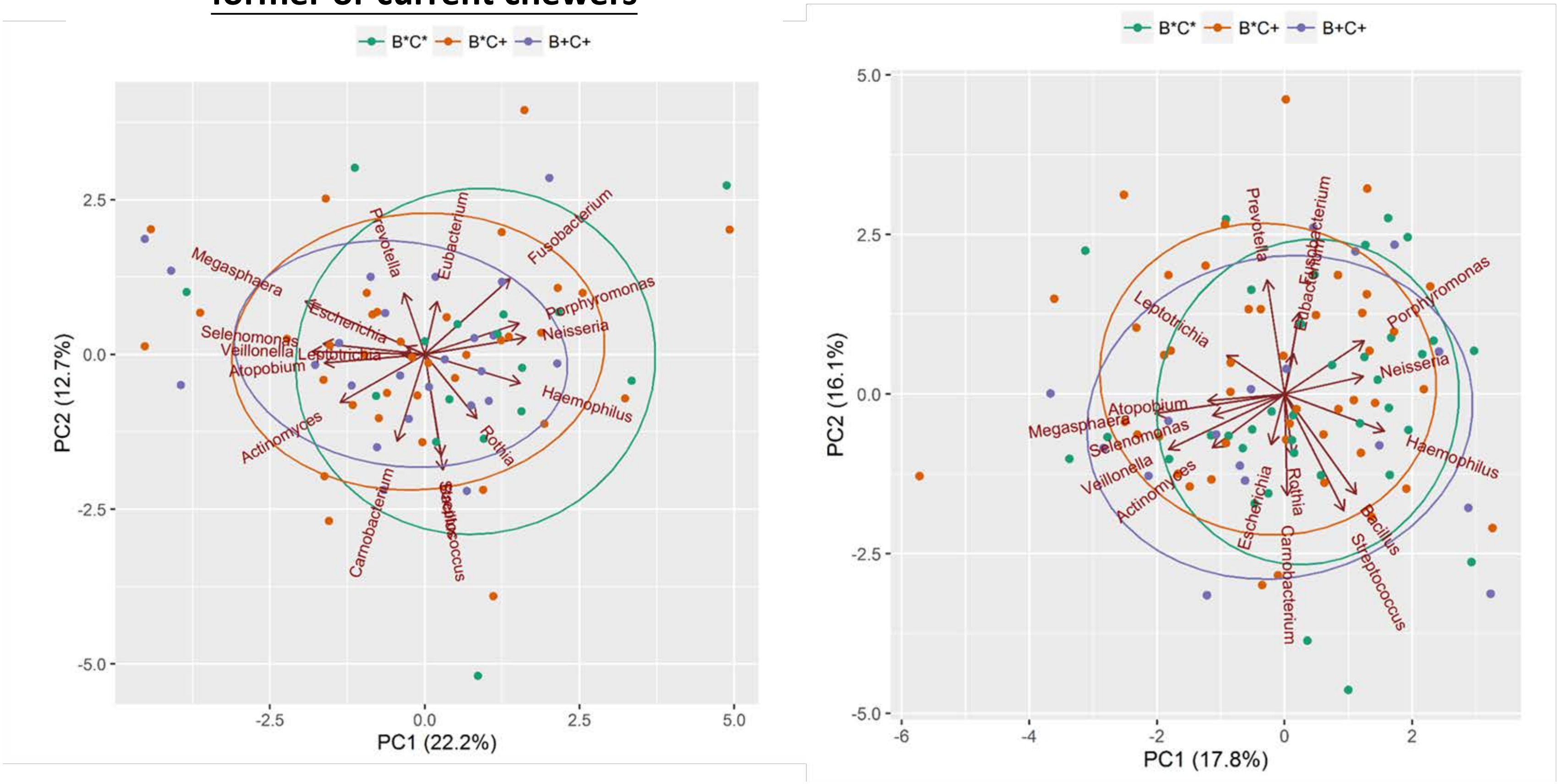

Figure S8. Principal component analysis of (log2-transformed) the top 17 genera for former or current chewers in the Epithelial precursor lesion and Cancer groups. The two first components (PC1 and PC2) were plotted. Individuals are represented by points. The length of the brown vectors represents the effect of each component on the status. The distance between them indicated their correlation. Circles represent three clusters formed because of close correlations. There was no separately clustering among subgroups.

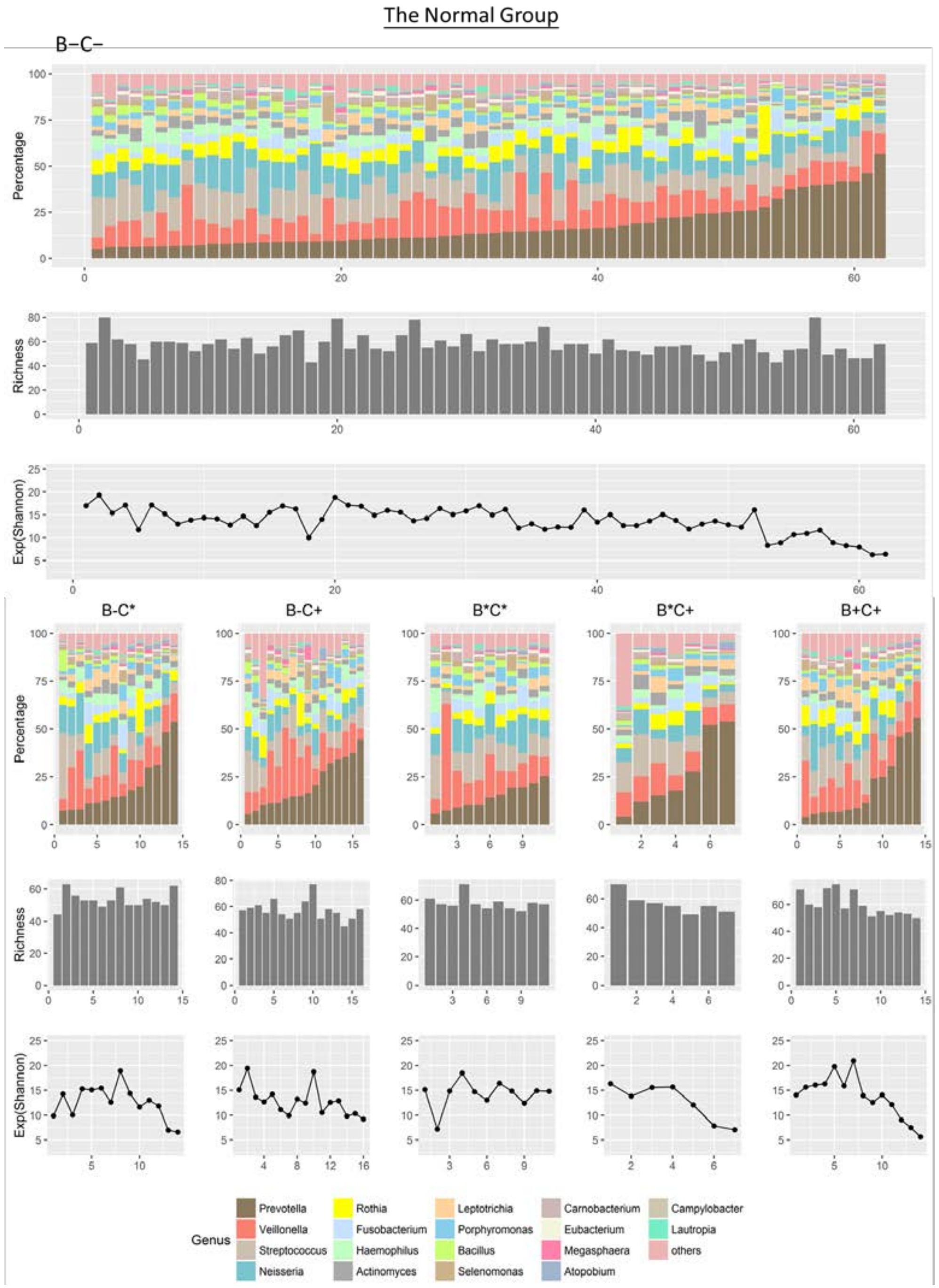

Figure S9. The composition of salivary microbiota within individuals of the Normal group. The salivary microbiota composition profiles were at the genus level revealed by 16S rRNA sequencing. Each color represents one bacteria genus. The top six genera within most individuals were *Prevotella*, *Veillonella*, *Streptoccus*, *Neisseria*, *Rothia*, *and Fusobacterium.* The distributions differed slightly within individuals, sorted by increasing *Prevotella*.

The Epithelial precursor lesion Group

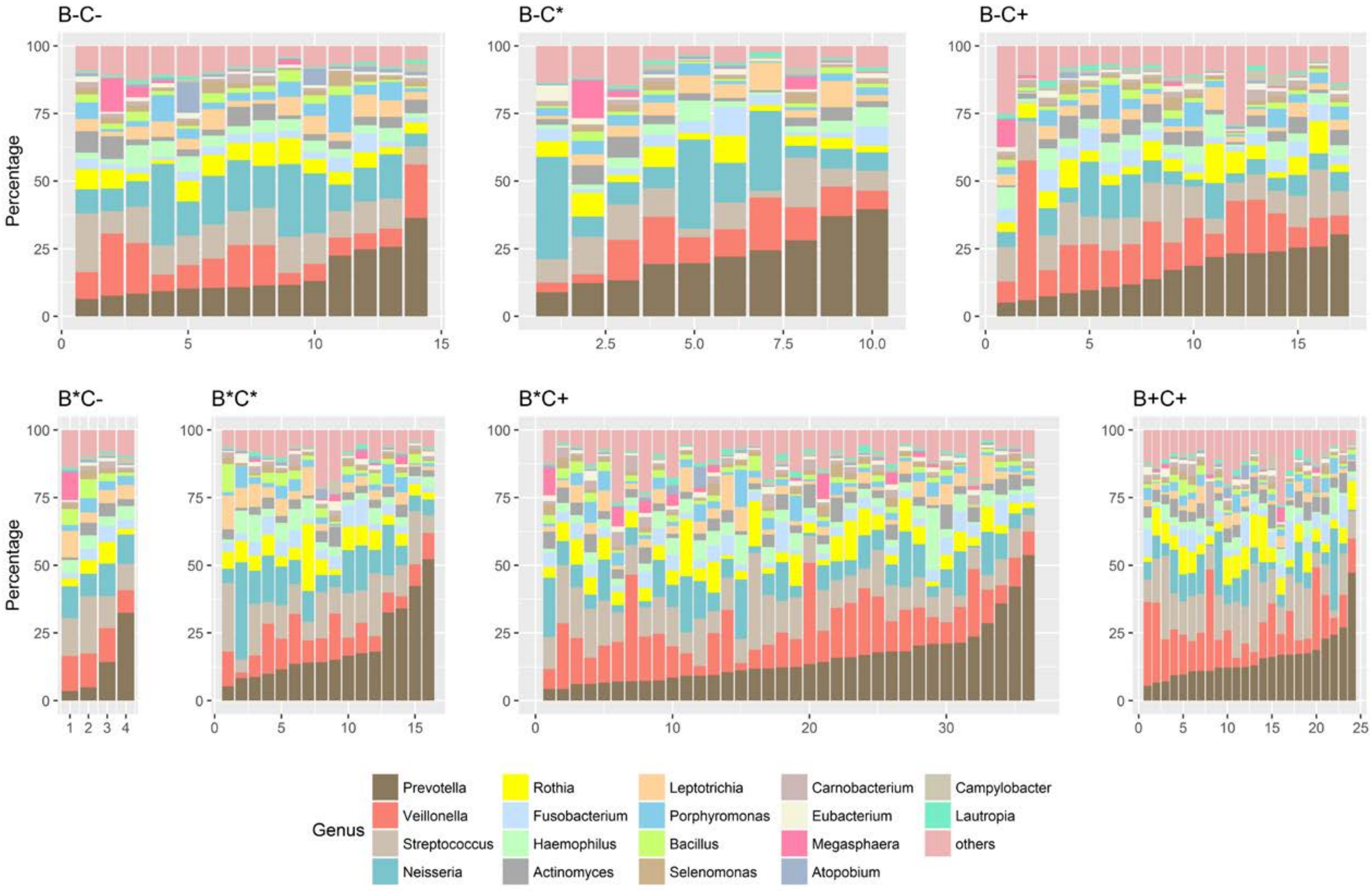

Figure S10. The composition of salivary microbiota within individuals of the Epithelial precursor lesion group. The salivary microbiota composition profiles were at the genus level revealed by 16S rRNA sequencing. Each color represents one bacteria genus. The top six genera within most individuals were *Prevotella*, *Veillonella*, *Streptoccus*, *Neisseria*, *Rothia*, *and Fusobacterium.* The distributions differed slightly within individuals.

The Cancer Group

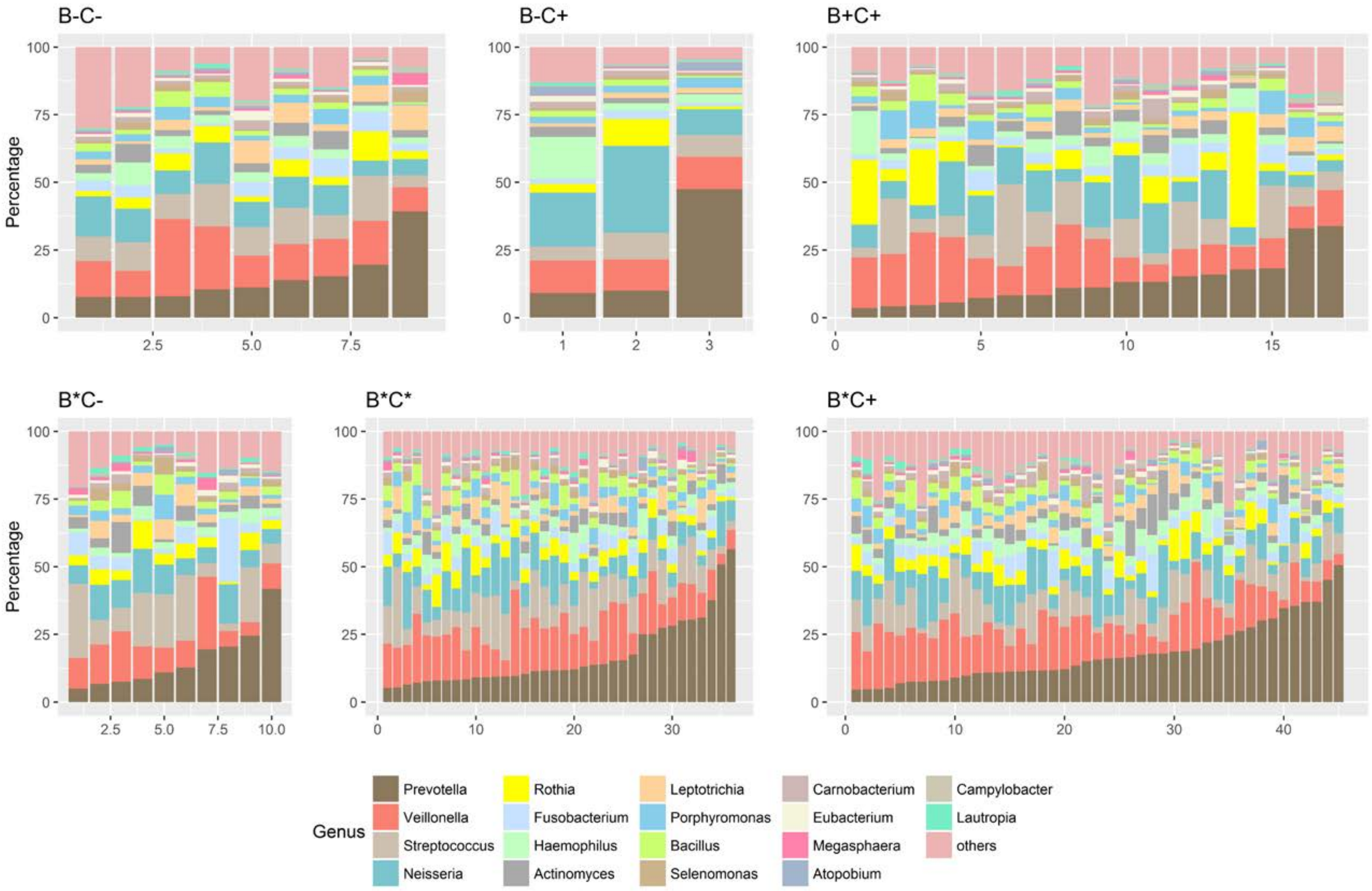

Figure S11. The composition of salivary microbiota within individuals of the Cancer group. The salivary microbiota composition profiles were at the genus level revealed by 16S rRNA sequencing. Each color represents one bacteria genus. The top six genera within most individuals were *Prevotella*, *Veillonella*, *Streptoccus*, *Neisseria*, *Rothia*, *and Fusobacterium.* Their distributions differed slightly within individuals.

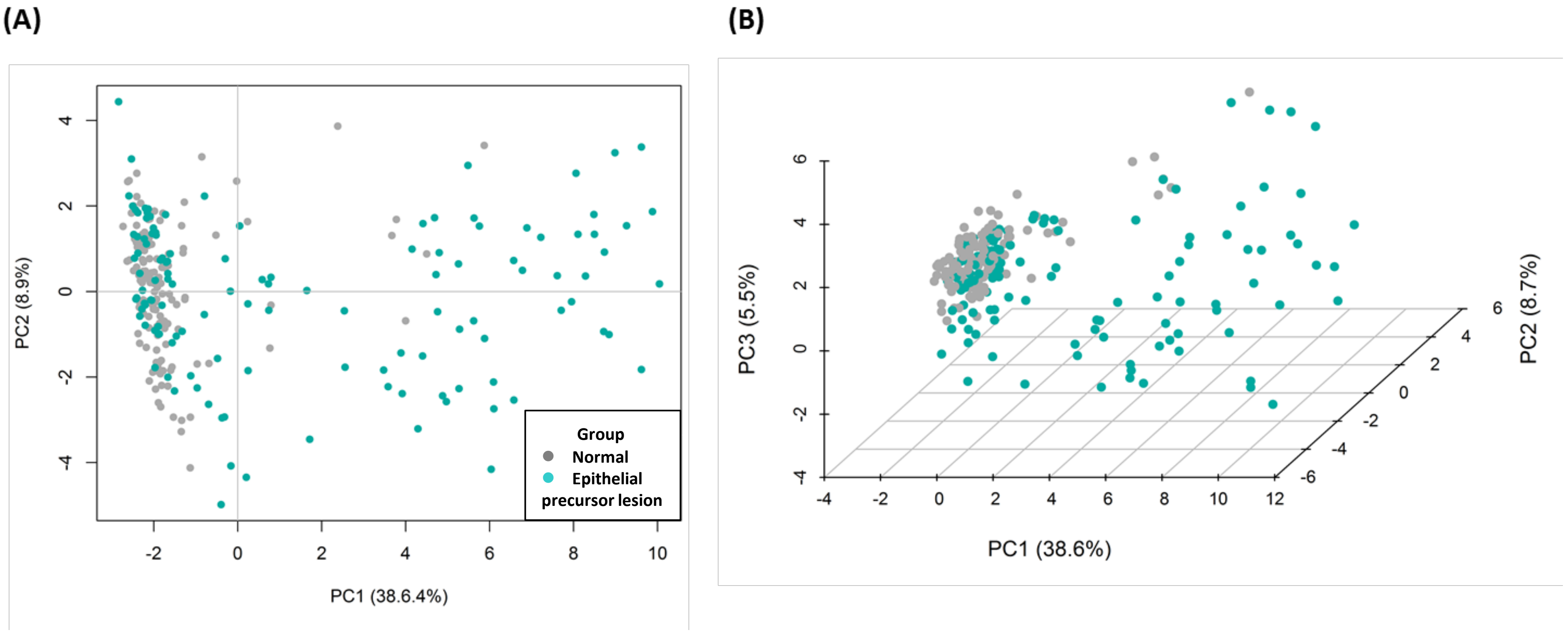

Figure S12. A Principal component analysis for 31 genera. The 31 genera revealed significant differences between the Normal and Epithelial precursor lesion groups. Patients with epithelial precursor lesions were separated into two parts. Points represent individuals.

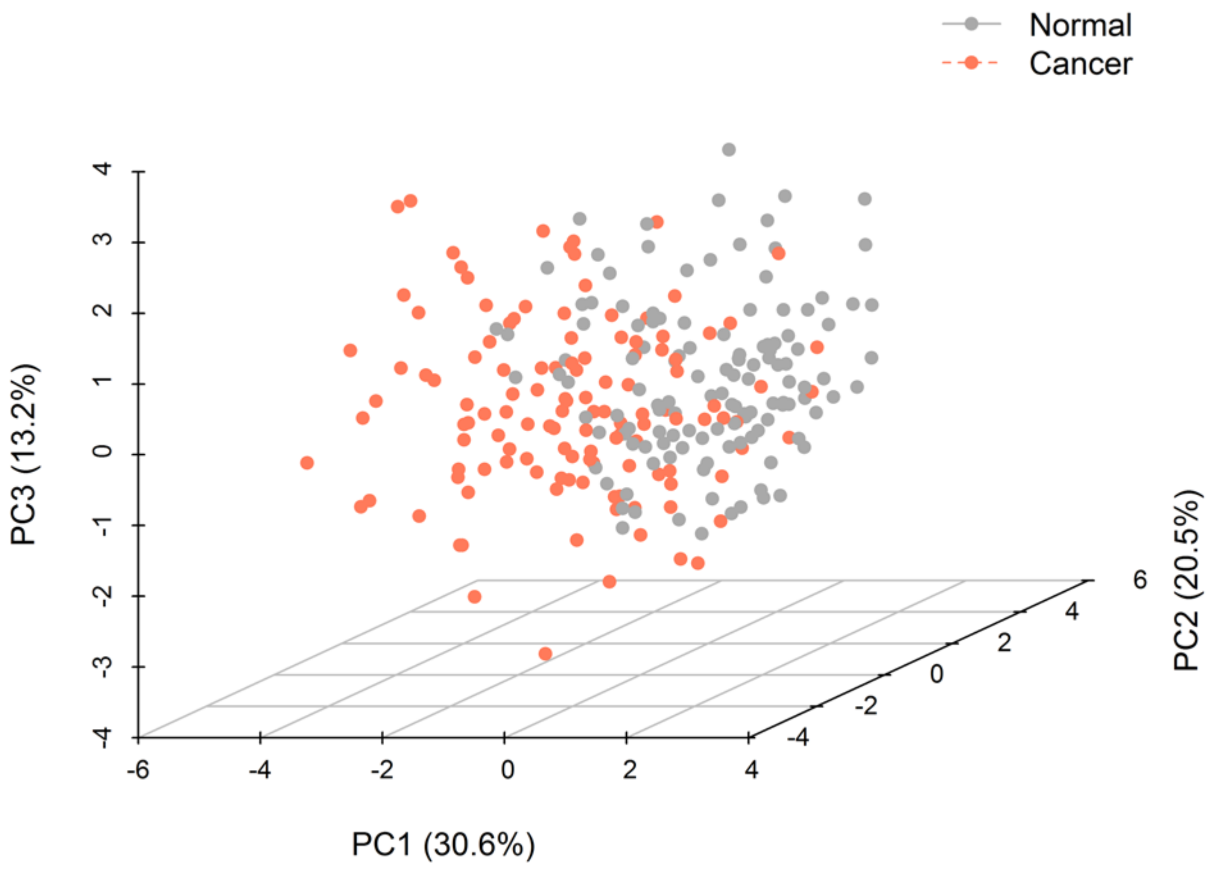

Figure S13. **A Principal components analysis for selected 10 genera.** The ten genera— *Bacillus*, *Bacteroides*, *Bifidobacterium*, *Clostridium, Escherichia, Filifactor, Peptostreptococcus, Schwartzia, slackia and Veillonella*— revealed significant differences between the Normal and Cancer groups, and these genera seemed to classify roughly patients into two clusters. Points represent individuals.

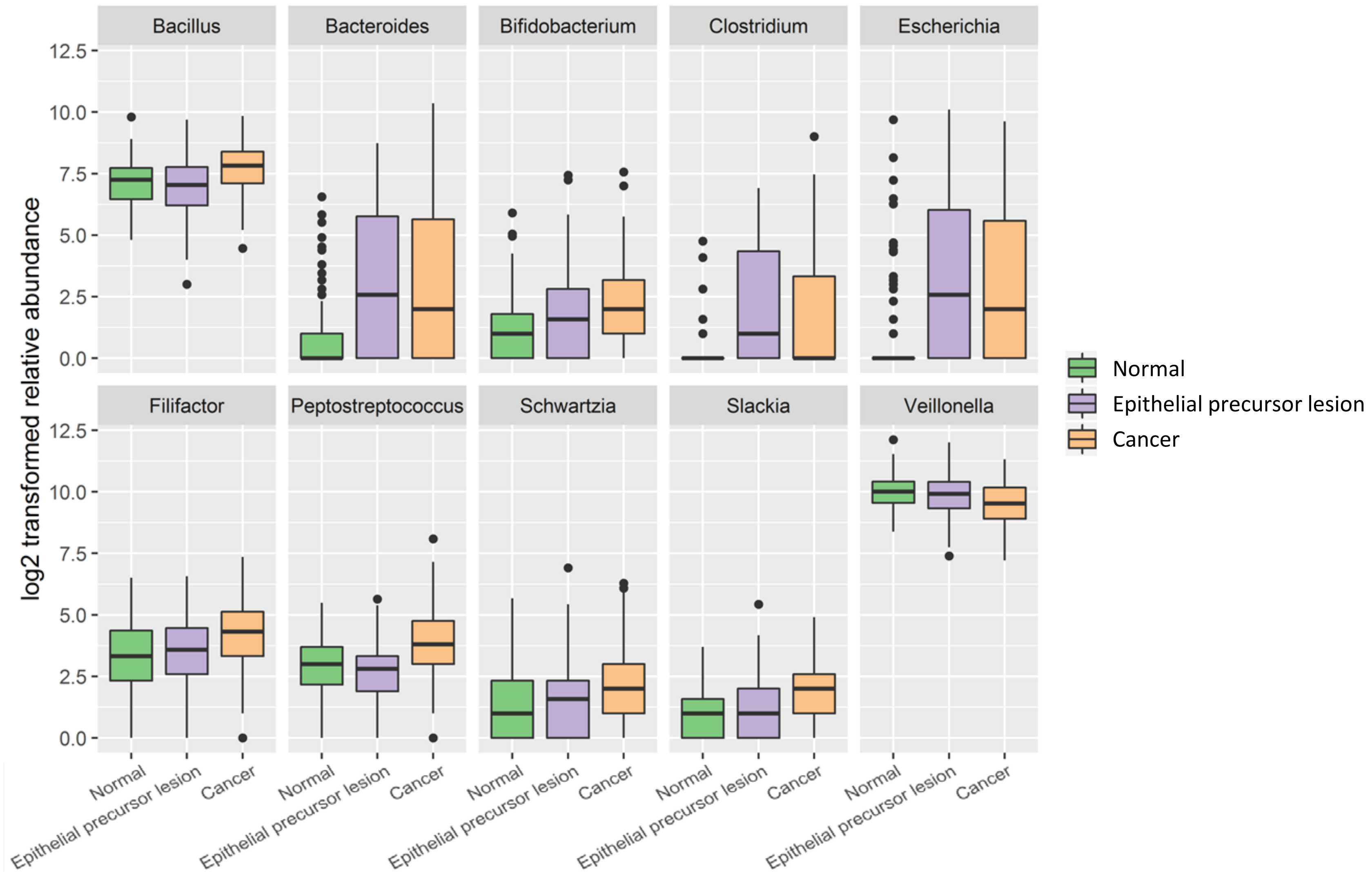

Figure S14. Boxplots of relative abundance for selected genera among the Normal, Epithelial precursor lesion, and Cancer groups. The ten genera— *Bacillus*, *Bacteroides*, *Bifidobacterium*, *Clostridium, Escherichia, Filifactor, Peptostreptococcus, Schwartzia, slackia and Veillonella*— revealed significant differences between the Normal and Cancer groups. The percentage of samples present *Escherichia* or *Clostridum* in the Normal group was only 13% and 14% , respectively . The others were present more than half of saliva samples.

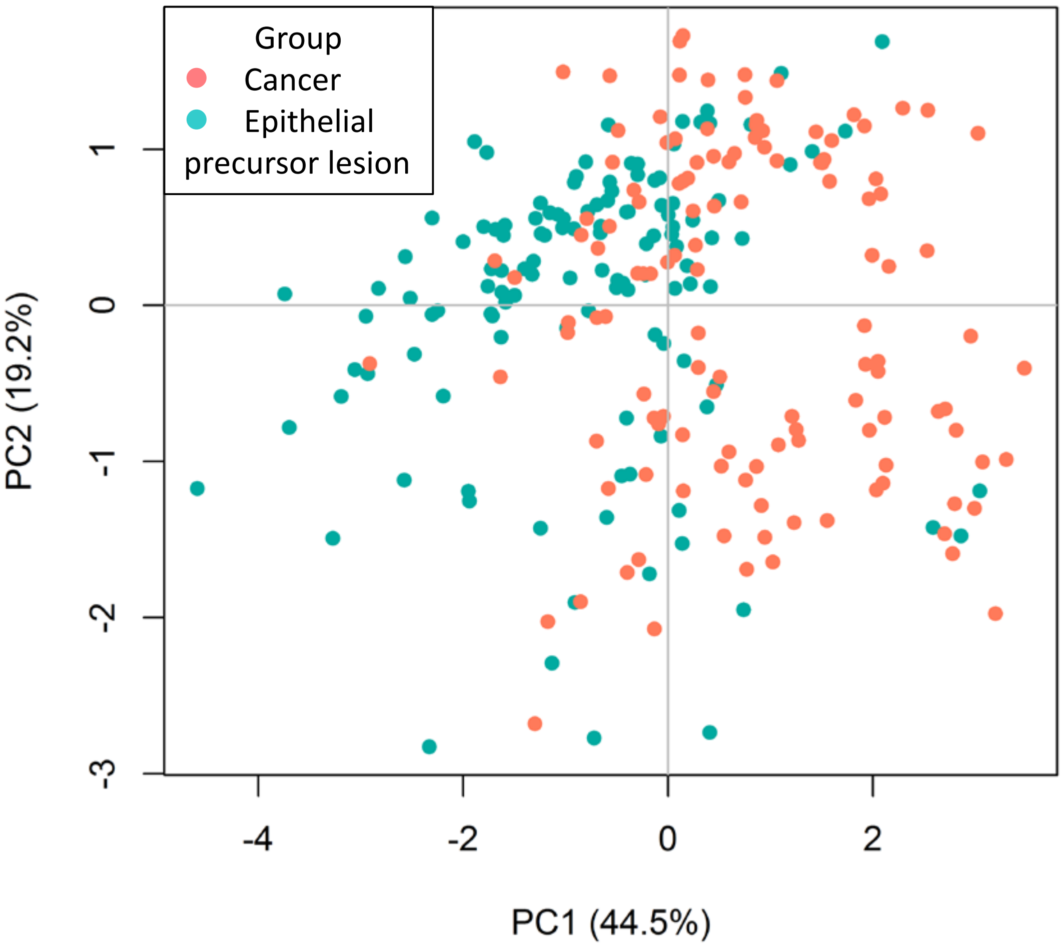

Figure S15. A Principal components analysis for five genera: *Bacillus*, *Enterococcus*, *Parvimonas*, *Peptostreptococcus*, and *Slackia*. The five genera—*Bacillus*, *Enterococcus*, *Parvimonas*, *Peptostreptococcus,* and *slackia*—revealed significant differences between the Epithelial precursor lesion and Cancer populations, and these genera seemed to classify roughly patients into two clusters. Individuals were represented by points.
